# Isolation of human monoclonal antibodies from 4CMenB vaccinees reveals PorB and LOS as the main OMV components inducing cross-strain protection

**DOI:** 10.3389/fimmu.2025.1565862

**Published:** 2025-04-16

**Authors:** Giacomo Vezzani, Viola Viviani, Martina Audagnotto, Alessandro Rossi, Paolo Cinelli, Nicola Pacchiani, Chiara Limongi, Laura Santini, Fabiola Giusti, Sara Tomei, Giulia Torricelli, Elisa Faenzi, Chiara Sammicheli, Simona Tavarini, Adriana Efron, Alessia Biolchi, Oretta Finco, Isabel Delany, Elisabetta Frigimelica

**Affiliations:** ^1^ GSK Vaccines, Siena, Italy; ^2^ Department of Pharmacy and Biotechnology (FABIT), University of Bologna, Bologna, Italy; ^3^ Departamento de Bacteriología, Instituto Nacional de Enfermedades Infecciosas-ANLIS “Dr. Carlos G. Malbrán”, Buenos Aires, Argentina

**Keywords:** 4CMenB, outer membrane vesicles, PorB, lipooligosaccharides (LOS), cross-strain protection, human monoclonal antibodies

## Abstract

**Introduction:**

The 4CMenB vaccine licensed against serogroup B Neisseria meningitidis (MenB) contains three recombinant proteins and Outer Membrane Vesicles (OMV) from a New Zealand epidemic strain. The protective response mediated on differentmeningococcal strains has been historically ascribed to one of the four main vaccine antigens fHbp, NHBA, NadA, and PorA nominated as the immunodominant antigen of the OMV component. It is however accepted that the extensive cross-protection observed after vaccination may be attributed to other proteins in the OMV. Here we interrogate the B cell responses elicited in humans to the OMV component after 4CMenB vaccination to elucidate the contribution of additional OMV antigens to meningococcal cross-protection.

**Methods:**

Following the isolation of plasmablasts from vaccinees, the OMV-specific human monoclonal antibodies (HumAbs) were recombinantly expressed and characterized for their binding and functional activity on a panel of MenB strains. Their target specificity was assessed through a tailor-made protein array and Western blot.

**Results:**

We found that 18 HumAbs showing bactericidal activity were PorB-specific, 1 was LOS-specific and 4 functional HumAbs remain with unknown targets. We identified three functional classes within the PorB HumAbs, through binding and in silico docking experiments, likely to be elicited from distinct epitopes on PorB and highlighting this antigen as a multi-epitope immunogenic OMV component responsible for distinct cross-protection across multiple MenB strains. Interestingly three of the PorB HumAbs and the LOS-specific HumAb showed bactericidal activity also against gonococcus.

**Discussion:**

We identified PorB and LOS as antigens on the OMV that may be implicated in the real-world observations of moderate protection against gonorrhea infection after OMV-based vaccinations.

## Introduction

Over the last three decades, several outer membrane vesicle (OMV)-based vaccines have been used to control infection from outbreaks of *Neisseria meningitidi*s serogroup B (MenB) causing meningococcal disease in Cuba (1), Norway (2), Chile (3), and New Zealand (4). Furthermore, one of the two broadly cross-protective MenB vaccines, currently licensed for all age groups, is the 4-component multivalent 4CMenB vaccine comprising detergent extracted OMV from the New Zealand 98/254 epidemic strain and three recombinant proteins (fHbp, NadA and NHBA), identified with the reverse vaccinology approach (5). The main OMV antigen contributing to protection has been historically recognized to be the Porin A (PorA), which is one of the most abundant proteins in the OMV (6) however non-PorA antigens are thought to contribute to the full extent of cross protection. Clinical studies have shown added value of inclusion of the OMV component with the three recombinant antigens in 4CMenB vaccine formulations for broad protection across MenB strains (7–9). Moreover, studies performed in infants showed that 4CMenB was able to induce immunogenicity and bactericidal activity against strains bearing heterologous PorA, suggesting that additional, non-PorA, antigens in the OMV could generate functional antibodies (8).

Due to the diversity of disease-causing isolates, the Meningococcal Antigen Typing System (MATS) was developed in an attempt to predict which strains would be covered by the multicomponent vaccine-induced responses. MATS combines conventional genotyping of PorA with a specialized sandwich ELISA that measures the levels of expression of fHbp, NadA, and NHBA proteins in a given meningococcal isolate and their immunological cross reactivity with the corresponding vaccine antigen (10). The effectiveness of 4CMenB vaccine measured after widespread implementation revealed that vaccine efficacy of 4CMenB in infants, measured as bactericidal activity elicited in infant sera, was usually higher than the strain coverage rates predicted using MATS (11, 12). Several non-exclusive explanations have been proposed to support the observed protection (1): synergy among antibodies targeting the multiple components included in the vaccine; (2) an intrinsic adjuvating effect of OMV and its components; or (3) the role of non-PorA antigens within the OMV component which act as additional protective antigens (13). Preclinical evidence on the importance of other OMV components in eliciting protective immune responses came from the work of Matthias and co-workers, which showed that OMV derived from bacteria depleted of PorA still conferred cross-strain protection in immunized mice and rabbits (14). Furthermore, after the MenZB immunization program in New Zealand, a vaccine that consists solely of the OMV component of 4CMenB, effectiveness against non-strain specific group B demonstrated protection beyond the PorA subtype (15). Finally, a recent publication from Viviani and collaborators identified OpcA and PorB as antigens involved in the broad cross-protection induced by the 4CMenB vaccine in mice and humans (16).

Besides the coverage of different MenB strains, 4CMenB elicits immune responses effective against non-B meningococcal serogroups that can be mainly attributed to antibodies targeting one or more antigens acting alone or synergistically (17–19). In particular, the potential coverage of 4CMenB on meningococcal serogroups A (20), C, W, X (21) and Y has been evaluated by testing a large number of clinical isolates from different countries in human serum bactericidal assay (hSBA). In addition, real world data suggest an effective role of 4CMenB vaccination in the prevention of infections from MenW strains (22–25).

Interestingly, a moderate effectiveness against gonorrhea infection has been reported recently in a number of observational retrospective studies after vaccination with OMV-containing meningococcal vaccines (26–33). Meningococcus and gonococcus, although etiological agents of very different human diseases, share greater than 85% of sequence conservation in their genomes (34) and it has been shown that OMV-induced antibodies recognized gonococcal proteins (35, 36) and human 4CmenB vaccinees have antibodies that recognize both gonococcal proteins and LOS (37, 38).

Overall, in the last decade the protective role played by different antigens contained in the OMV component of 4CMenB vaccine has been demonstrated, but their exact identity and contribution still need to be fully understood and characterized. Interrogating vaccine-induced B cell responses in humans is a powerful approach to identify protective antigens and epitopes included in a vaccine formulation. The approach, named Reverse Vaccinology 2.0, is based on the isolation of the variable regions of heavy (VH) and light (VL) chain genes of vaccine-specific immunoglobulins and on their recombinant expression and functional characterization (39, 40). This approach has been previously applied to isolate and characterize human monoclonal antibodies (HumAbs) elicited by the three recombinant proteins of 4CMenB in adult subjects after vaccination (5). Very recently the isolation of gonococcal -specific HumAbs from memory B cells from 4CMenB vaccinees has been reported (Troisi, Fabbrini et al., 2023, bioRxiv, https://www.biorxiv.org/content/10.1101/2023.12.07.570438v1). Here we selected vaccinees that after 4CMenB immunization are highly responsive to the OMV component and recovered the Ig sequences from single sorted B cells, to clone HumAbs from vaccine-responsive plasmablast mononucleate cells (PBs). By characterizing the OMV-specific HumAbs, we determined the antibody targets and hence immunogenic antigens contained in the OMV preparation. We characterized their bactericidal killing against a panel of meningococcal strains as well as their cross-functionality on *N.gonorrhoeae*.

## Methods

### Ethics statement

Human samples obtained from adults immunization in a Phase I clinical study conducted in Krakow, Poland and sponsored by Novartis Vaccine, now part of the GSK group of Companies, using two doses of multicomponent serogroup B meningococcal vaccine 4CMenB formulations. In this study vaccinated subjects were immunized with formulations with 2 different doses of OMV component: rMenB + 25 µg OMV, and rMenB + 6.25 µg OMV. The Clinical trial protocol was approved by the Bioethics Committee of the District Medical Doctors’ Chamber in Krakow (authorization number: 87/KBL/OIL/2010; approval date: September 15^th^ 2010) and the study was conducted in accordance with the Declaration of Helsinki. Written informed consent was obtained from each of the subjects.

### Elispot assay

Plasmablasts were collected from subjects 1 week after the second immunization and were tested for specificity and quantity using a standard ELISpot protocol. Ninety-six well ELISpot plates (Millipore MultiScreenHTS HA Filter Plate) were coated with 100 µL/well of phosphate-buffered saline (PBS), OMV (5 µg/mL) or 2.5 µg/mL goat anti-human IgG +5 µg/mL goat anti-human IgM (BD Pharmingen). Coated plates were incubated at 4°C overnight and then washed 3 times with 200 μl per well of sterile PBS. Wells were blocked with 200 ul of PBS containing 1% dried skimmed milk for 2 h at room temperature prior to the addition of cells, which were diluted in complete medium (RPMI, Invitrogen 12633012, supplemented with 5% FBS, HyClone, Cytiva SH30070.01HI). Suspensions of 4 − 8 × 10^6^ thawed PBMC were seeded in duplicate wells and serially diluted 2-fold in a final volume of 100 µl/well and plates were incubated at 37°C, 5% CO2 for 2 h before stopping the assay by extensive washing with PBS 0.05% - Tween 20 (Sigma–Aldrich). Secondary antibodies anti human IgG FITC (Jackson ImmunoResearch 609-095-213), and anti-human IgM biotinylated (BD Biosciences 314504) were then added for detection in 100 µl/well of PBS containing 4% bovine serum albumin and incubated ON at 4°C. After the incubation, plates were washed and further incubated for 40 min with 100 µl/well of PBS - 0.05% Tween20 containing horseradish peroxidase (HRP)-conjugated streptavidin (MERCK GERPN1231) and an alkaline phosphatase (AP)-conjugated mouse anti-FITC antibody (Roche 11426338910). Spots of antibody secreting cells were revealed by adding in the dark the HRP substrate AEC kit (Vectro Laboratories SK4200) for 40 min and, after extensive washings with deionized water, the AP substrate kit III for 40 min (Vector Laboratories SK5300). Antigen-specific and total IgG and IgM Ab-secreting cells were enumerated using the CTL immunospot S5 UV analyzer (CTL Europe, Bonn, Germany).

### Flow cytometry sorting of human B cells

Plasmablasts (PBs) were isolated from human peripheral blood cells. PB cells were stained with anti-human CD27-PE (BD Biosciences, 340425), CD38-Cy7 (BD Biosciences 335825), CD19-V421 (BD Biosciences 562440), CD20-FITC (BD Biosciences 345792) and IgD-A700 (BD Biosciences 561302) for 30’ at 4°C. After washing with 1% FBS in PBS, cells have been centrifuged and resuspended in PBS/EDTA 2.5 mM. PB subset has been identified as CD19+CD20dmIgD-CD27++CD38++ and single-cell sorted using a FACS sorter (BD Biosciences, FACSAria III Cell Sorter) into 96w plates containing lysis buffer (RNAse OUT, Invitrogen 10777019; BSA, AMBION AM2616 1 mg/ml in DEPC water, AMBION AM9915G) and immediately frozen for storage at -80° until use.

### Cloning of variable region genes and HumAb recombinant expression

Heavy and light chain variable (VH and VL, respectively) region genes of single plasmablasts isolated from peripheral blood were retrotranscribed with gene-specific primers and amplified separately by 2 nested PCR reaction, performed with a mix of primers designed to amplify the highest number of V families (**Supplementary Table 1**), and then ligated by Polymerase Incomplete Primer Extension (PIPE) into mammalian expression plasmids (pcDNA 3.1, Thermofisher V79020) containing the leader sequence for secretion and the constant region fragment of IgG1 heavy or k-light chain, respectively. The plasmid carries, within the coding sequence of the human IgG1 constant region of heavy chain, the hexabody mutations E345R/E430G or E345R/E430G/S440Y to enhance functionality of obtained mAbs (41). For transient expression of mAbs, two separate linear Transcriptionally Active PCR (TAP) products were generated for each paired VH-VL ligation product. TAP fragments were amplified from the plasmid ligation reactions of the variable regions with primers annealing on the plasmid and included the HCMV promoter, the full antibody sequence and the plasmid poly-adenylation signal (protocol adapted from (42)). The paired TAP products were then used for co-transfection and transient expression in Expi293F cells (Thermofisher A14527), using Expifectamine (Thermofisher A14525) according to manufacturers’ instructions.

### HumAb purification from culture supernatants

The sequences corresponding to VH and VL of desired human monoclonal antibodies were obtained by Sanger and Illumina sequencing of the TAP products, codon-optimized for mammalian expression and synthetized either by GeneArt (Thermofisher) or Twist and cloned into expression vectors containing the human IgG1 hexabody, Igκ or Igλ constant regions. Transient recombinant expression of antibodies was achieved by co-transfecting paired heavy and light chain expression plasmids into suspension cultures of Expi293F cells according to manufacturer’s protocol. Supernatants were collected 6 days after transfection. Culture supernatants were recovered after centrifugation at 900×g for 10 min and filtered through a 0.22 µm pore size filter (Millipore P1313), and recombinant antibodies were purified by affinity chromatography with Protein G (GE Healthcare 17061802) according to the manufacturer’s instructions. Antibodies were eluted with 0.1 M glycine (pH 2.1), neutralized in 1 M Tris (pH 8.0) and then the buffer was exchanged to PBS. Purified antibodies were quantified by absorbance at 280 nm and their purity was assessed by SDS-page electrophoresis and ProBlue safe staining (Giotto Biotech, G00PB005).

### Luminex binding screening of HumAbs in culture supernatants

For IgG quantification and binding specificity of the raw supernatants from TAP-transfected Expi293 cells, a Luminex assay was developed. The vaccine components fHBP-GNA2091, GNA1030-NHBA, and NadA recombinant antigens, and OMV from NZ98/254 (43), and the recombinantly expressed PorA (16) and Protein A were individually coupled to 6 distinct luminex beads (MC12XXX; MagPlex) according to the manufacturers’ instructions. Coated beads were incubated for 1 hour with 5 µl of raw supernatant diluted (1:2) in PBS with 0.05% Tween 20 (Sigma-Aldrich 28320) and 1% bovine serum albumin (BSA, A3294 Merck). After washing in PBS with 0.05% Tween 20, the beads were incubated for 45 min with R-Phycoerythrin-AffiniPure F(ab’)2 Fragment Goat Anti-Human IgG, Fcγ Fragment Specific (109-116-098 Jackson Immunoresearch) and signals were acquired with BioPlex200 (BIO-RAD) (Subjects 1 and 2) or with BioPlex 3D suspension array system (BIO-RAD) (Subject 3). All fluorescence intensities were subtracted of background signals, represented by the signal of the beads incubated with a transfection supernatant of an unrelated mAb or a supernatant of non-transfected cells. A standard curve with human IgG was made to extrapolate the mAbs concentration in the supernatants with a dynamic range between 1 ng/ml and 50 ng/ml. All signals were analyzed with Bio-Plex manager software (BIO-RAD). Supernatants with estimated IgG concentration ranging between 10 and 20 ng/µl and with an antigen-specific MFI greater than 5000, or with estimated IgG concentration greater than 20 ng/µl and an antigen-specific MFI greater than 10000, were deemed positive for that antigen. All supernatants analyzed with BioPlex 3D instrument showing antigen-specific MFI greater than 1000 were deemed positive for that antigen. With both instruments, in case of MFI < 1000, supernatants were deemed positive if relative concentration was <10 ng/µl (**Supplementary Table 2**).

### Bacterial strains and growth conditions

The meningococcal and gonococcal strains used in this study are listed in **Supplementary Tables 3** and **4** respectively. Bacteria were routinely grown overnight at 37°C on plates containing GC media with CO_2_ for meningococcus strains, or on plates containing GC with 1% IsoVitaleX (BD Biosciences, 11798163) for gonococcal strains. Unless differently stated, the liquid growth conditions were the following: meningococcal bacteria were grown in Mueller-Hinton Broth (MHB) containing 0.25% (w/v) glucose until early log phase (OD_600_ of ~ 0.25). *N.gonorrhoeae* (*Ng*) strain FA1090 bacteria were grown at 37 °C in GC liquid medium supplemented with 1% IsoVitaleX and 1 µg/mL of CMP-NANA (Cytidine-5′-MonoPhospho-N-Acetyl NeurAminic acid sodium salt, Merck C8271) until mid-exponential phase.

### OMV production and purification

*N.meningitidis* and *N.gonorrhoeae* strains were plated on GC agar plates or GC +1% IsoVitaleX, respectively. Plates were incubated overnight at 37°C in 5% CO_2_. The following day, *N. gonorrhoeae* colonies were inoculated in 5 ml of GC + 1% IsoVitaleX + lactate and the growth was maintained for 28 h in 24 deep-well plate at 37°C with shaking at 350 rpm. *N.meningitidis* colonies were instead inoculated in 10 ml of Mueller-Hinton Broth at a starting optical density at 600 nm (OD_600_) of ∼0.05 and grown until OD_600_ of ∼1.0-1.5 at 37°C. Then 10 ml were transferred in 50 ml of prewarmed slightly modified MCDMI medium and incubated at 37°C in 5% CO_2_. OD_600_ was constantly monitored, and the growth was stopped when OD_600_ remained stable for 1.5 hours. Bacteria cultures were clarified by centrifugation for 60 min at 4000xg and the supernatants were subjected to high-speed centrifugation at 119000xg for 2 h at 4°C (Beckman Coulter Optima Ultracentrifuge). The pellets containing the OMV were washed with phosphate buffer saline (PBS), ultracentrifuged again as above and finally resuspended in PBS. OMV total protein content was quantified through the Lowry assay (DC Protein Assay, BioRad) following manufacturer’s instructions.

### Protein array design, generation, validation and hybridization

All the HumAbs produced and purified were tested for antigen identification on protein microarrays previously generated (16). In particular, the recombinant protein microarray encompassed 12 recombinant proteins spotted at 0.5 mg/ml in 40% glycerol along with the three recombinant meningococcal antigens of the 4CMenB vaccine (NHBA-GNA1030; GNA2091-fHbp and NadA at 0.5 mg/ml in 40% glycerol) while the vesicles protein chip contained 26 recombinant *E.coli* Generalized Modules for Membrane Antigens (GMMA) expressing meningococcal antigens and two GMMA empty (16) (**Supplementary Figure 3A**). The species-specific *N.meningitidis* and *N.gonorrhoeae* OMV array were printed with 18 different meningococcal (at 1.0 mg/ml or 0.5 mg/ml in 20% glycerol) and 23 gonococcal OMV (at 0.25 mg/ml in 20% glycerol). Controls consisted of 8 serial two-fold dilutions of human IgG (from 0.5 mg/ml to 0.004 mg/ml in 40% glycerol), unrelated proteins expressed and purified from *E. coli* following the same expression and purification protocol but originating from pathogens other than MenB (0.5 mg/ml in 40% glycerol), and PBS + 40% glycerol spots. Each sample was spotted randomly in replicates per array onto ultra-thin nitrocellulose coated glass slides (FAST slides; Maine Manufacturing Z721158). Printing was performed with the ink-jet spotter Marathon Argus (Arrayjet) (200 pl each spot) in a cabinet with controlled temperature and humidity (18 °C and 50–55%, respectively). To ensure efficient and reproducible protein immobilization a preliminary array validation was carried out. Preliminary experiments with mAbs showed that a range of 0.5-1 µg/ml corresponded to the best signal to noise ratio. For mAbs hybridization experiments, nonspecific binding was minimized by preincubating the slides with a blocking solution (BlockIt, ArrayIt BKT) for 1 hour. Purified mAbs were then diluted in Block-It buffer and overlaid for 1 h at room temperature prior to undergoing two washes with Tween 0.1% in PBS (TPBS). AlexaFluor 647-conjugated anti-human IgG secondary antibody (Jackson Immunoresearch, 115-605-174) diluted 1:800 was incubated for another hour, before proceeding with slide scanning. Fluorescence images were obtained using InnoScan 710 AL (Innopsys) and the images were generated with Mapix software at 10 μm/pixel resolution. ImaGene 9.0 software (Biodiscovery Inc.) was used to calculate spot fluorescence intensities while the microarray data analysis step was carried out with an in-house developed R script. For each protein the Mean Fluorescence Intensity (MFI) of replicates was obtained after the subtraction of local background values surrounding each spot. MFI greater than 5000, corresponding to the MFI of control protein spots after detection with fluorescent-labelled antibodies, plus ten times the standard deviation, were considered positive. MFI scores were ranked in four categories (1): high reactivity; MFI ≥ 30000; (2) medium reactivity; 15000 ≤ MFI < 30000; (3) low reactivity; 5000 ≤ MFI < 15000; (4) no reactivity; MFI < 5000.

### Bactericidal activity assay

The bactericidal activity of the HumAbs against *N.meningitidis* strains was evaluated in a bactericidal assay with rabbit complement as previously reported (5). Briefly, meningococcal bacteria were grown in MHB containing 0.25% (w/v) glucose until early log phase (OD_600_ of ~ 0.25) and diluted in Dulbecco’s Phosphate Buffered Saline (DPBS) containing 0.25% glucose and 0.1% BSA to the working dilution of 10^5^ CFU/ml and incubated with serial two-fold dilutions of test monoclonal antibodies starting from either raw supernatants from TAP transfections diluted 1:4, or purified HumAbs at a concentration of 125 µg/ml with the addition of 25% baby rabbit complement (Cedarlane CL3441-R). Bactericidal activity of mAbs against *Ng* strain FA1090 was assessed with a similar approach. Bacteria were grown at 37 °C in GC liquid medium supplemented with 1% IsoVitaleX and 1 µg/mL of CMP-NANA until mid-exponential phase. Then bacteria were diluted in Dulbecco’s Phosphate-Buffered Saline (DPBS, Sigma) containing 0.1% glucose and 1% BSA, to the working dilution of 1x10^3^ CFU/ml and incubated for 1h at 37°C with serial two-fold dilutions of test monoclonal antibodies and exogenous human complement, obtained from volunteer donors under informed consent, at 10% final concentration. After the incubation, 100 µl of GC medium plus 0.5% of Bacto Agar was added to the reaction mixture and incubated overnight at 37C° with 5% CO2. The day after, the plate well images were automatically acquired with a high throughput image analysis system and the Colony Forming Units (CFUs) were automatically counted for each well by an internally customized colony counting software. Bactericidal titers were defined as the monoclonal antibody concentration giving 50% decrease in CFU number compared to the reaction mixture, in the absence of antibodies.

### LOS and protein immunostaining

To analyze LOS, meningococcal strains were grown in the same conditions utilized to perform bactericidal assay experiments. Once OD_600_ of ~0.25 was reached, 30 ml of culture were centrifuged at 2500xg for 15 minutes, washed with sterile-filtered PBS, and pelleted again. Bacterial pellets were boiled for 10 minutes at 100°C in reducing conditions (1x 1,4-dithioreitol, Sigma Aldrich 15508013) and equilibrated in LDS sample buffer 1x (ThermoFisher J61894) and 10 µl were loaded into 4-12% Bis Tris gel that was run at 120 V for 90 minutes. To analyze the proteins, the same preparation and running procedures were applied to 10 µg of each protein of interest loaded in the wells. Proteins and polysaccharides were transferred on a nitrocellulose membrane with iBlot (Invitrogen IB23001) and membranes, after 1h saturation in PBS - 10% BSA, were incubated overnight at 4°C with the antibodies for which the target was to assess. After 3 washes with PBS - 0.5% Tween20 (PBST) membranes were incubated with anti-human HRP conjugated polyclonal antibody (Invitrogen A18817) for 1 hour at room temperature, washed three times with PBST and incubated for 5 minutes with Super signal west pico PLUS (ThermoFisher 34579). Chemiluminescent signals were acquired by ChemiDoc instrument (BIORAD) with auto optimal option.

### Electron microscopy immunogold experiments

Meningococcal strains were grown in the same conditions utilized to perform bactericidal assay experiments. Once OD_600_ of ~0.25 was reached, 5 ml of culture were centrifuged at 2500xg for 15 minutes, washed with sterile-filtered PBS, and centrifuged again. Pelleted bacteria were then resuspended in 4% paraformaldehyde for 5 minutes at room temperature for fixation. The fixation buffer was then removed, and bacteria resuspended in PBS to a final OD_600_ of 1. Five µl of bacteria suspension were adsorbed to 300-mesh nickel grids, blocked in PBS containing 1% bovine serum albumin (BSA) and incubated with HumAbs at a concentration of 0.5 µg/ml in PBS, for 1 h. Grids were washed several times and incubated with 12-nm gold-labeled anti-human secondary antibody diluted 1:40 in PBS for 1 h. After several washes with distilled water the grids were air dried and analyzed using a TEM FEI Tecnai G2 spirit microscope operating at 120kV. The micrographs were acquired using a Tvips TemCam-F216.

### FACS staining

Bacteria were pelleted at 2500xg for 15 minutes, washed and resuspended in an equal amount of sterile-filtered PBS. 50 µl of bacterial suspension were incubated for 1h at RT with 50 µl of each HumAb at a final concentration of 10 µg/ml. Bacteria were pelleted at 4000xg for 5 minutes, washed 2 times and resuspended in 100 µl of anti-Human IgG-FITC conjugated antibody (Invitrogen, cat. 31529) at the final concentration of 10 µg/ml diluted in PBS - 1% BSA - 0.5% Tween20. After 30 minutes incubation at room temperature, bacteria were washed twice, and fluorescent intensity was acquired by CANTOII Flow cytometer (Becton Dickinson). Signals were analyzed by FlowJo software (BD Biosciences), considering as positive bacteria with fluorescence intensity higher than the negative control, i.e. bacteria incubated only with secondary anti-Human IgG-FITC conjugated antibody.

### Functional data clustering

The mAbs clustering, based on functional data, was performed using *ad-hoc* code developed in Python (v3.9.12) by using the *AgglomerativeClustering* function from the Scikit-learn (v0.24.1) package (https://scikit-learn.org/). To express a global measure of similarity for the functional activity of the 18 tested anti-PorB mAbs, binding and killing activity data were combined. Since the two data sources presented very different scales, they were pre-processed separately to transform data in the same range. MFI values from binding activity were scaled by computing the logarithm (base 10) and then normalized in the range by the Min-Max normalization. To describe the bactericidal activity, we started from the last detected value of positivity from the serial two-fold dilutions of the assay. We applied a logarithmic scale, with base 2, to get the number of dilution steps and, again, we normalized data in the range by the Min-Max scaling. The two datasets (binding and killing activity) were then merged to create a unique set of normalized features for each mAb. The distance matrix was computed by the Euclidean distance and the threshold for the hierarchical clustering was set to fall in the largest interval where the number of clusters remains constant when varying the threshold itself.

### Sequence analysis and clustering

Sequence level properties of acquired HumAbs were analyzed after alignment against human Ig germlines downloaded from IMGT (https://www.imgt.org/) germline database (v202038-1). A first alignment of nucleotide sequences was performed by NCBI IgBLAST (v1.17.1) suite (44) to get V(D)J gene labeling and CDRs/FWRs regions annotation, together with the corresponding aminoacidic sequence translation. ANARCI (https://opig.stats.ox.ac.uk/webapps/newsabdab/sabpred/anarci/) was used to annotate equivalent antibody residue positions enabling the comparison of conserved amino acid residues in the hyper-variable CDRs loops following the canonical IMGT numbering scheme (45). Obtained annotations were used to cluster mAbs sequences in clonotypes having identical V-J genes and maximum 1 aa mismatch over 12 residues in CDR3 (46). We grouped sequences based on the heavy chain only, this one being usually assumed to contribute more to the epitope binding. All the data processing and clustering was performed in Python (v3.9.12) with the Scikit-learn (v0.24.1) package (https://doi.org/10.48550/arXiv.1201.0490). The Plotly (v5.9.0) package (https://plotly.com/) was used for visualization, while the clonotype network was realized with the NetworkX (v.2.6.3) package (https://networkx.org/).

### Computational structure modeling and docking experiments

PorB forms trimer on the bacterial surface as well as in the available X-ray structure (PDB: 3VZT). The whole trimeric organization was used in the docking set up but only the monomer unit was considered as binding partner. Similarly, only the variable domain of the antibody was docked. The New Zealand 98/254 MenB PorB 3D structure was modeled with AlphaFold2 multimer protocol showing an RMSD of 2.68 Å compared to the experimental structure publicly available (PDB: 3VZT) and highlighting the conformational variability of the loop regions. The HumAbs were modeled with DeepAb (47) by applying the default protocol.

We docked the 18 selected mAbs to the modeled trimeric PorB with version 2.4 of the HADDOCK software (48). The binding site on PorB was defined as the solvent-exposed loop regions with respect to the embedded transmembrane part. The paratope region of the selected mAbs was identified with Paragraph (49) and defined in HADDOCK as “active” while the epitope regions on PorB as “passive”, meaning the paratope region needed to make contact with at least one of the PorB residues and there was no penalty if it didn’t contact them all, allowing the HumAb to freely explore the binding loops. All three docking iterations (namely it0, it1, and water) were performed generating 1000, 400, and 200 poses respectively using the default values and scoring function. Clustering was performed based on backbone RMSD with a distance cutoff of 5 Å on the latest 200 generated poses. Finally, the lowest score was used to select the “best cluster” as the most antigen/antibody interaction representative.

## Results

### Isolation of plasmablasts from blood of 4CMenB vaccinees identifies 100 OMV-specific HumAbs

To interrogate the human B cell responses to the OMV component of 4CMenB, we screened Peripheral Blood Mononuclear Cells (PBMCs) from multiple subjects after vaccination with 4CMenB formulations for OMV-specific Plasmablasts (PBs). PBMCs from 56 adult subjects immunized with the 4CMenB vaccine formulations were collected 1 week after the last vaccine dose. The percentage of OMV-specific PBs in the total population of PBMCs from each subject was measured by ELISpot analysis. We observed some heterogeneity in the frequency of OMV-specific PBs among the different subjects, which ranged from more than 20% in a few subjects to less than 5% in half of the individuals (**Supplementary Figure S1**). We selected three subjects (Sbj 1-3) among those with the highest frequency of OMV-specific PBs for which adequate PMBCs were recovered for the following analyses. Interestingly, Sbj1 and Sbj3 were immunized with the complete formulation of 4CMenB vaccine (rMenB + 25 µg OMV), while Sbj2 received the formulation containing ¼ of the OMV dose (rMenB + 6.25 µg OMV).

**Figure 1:**
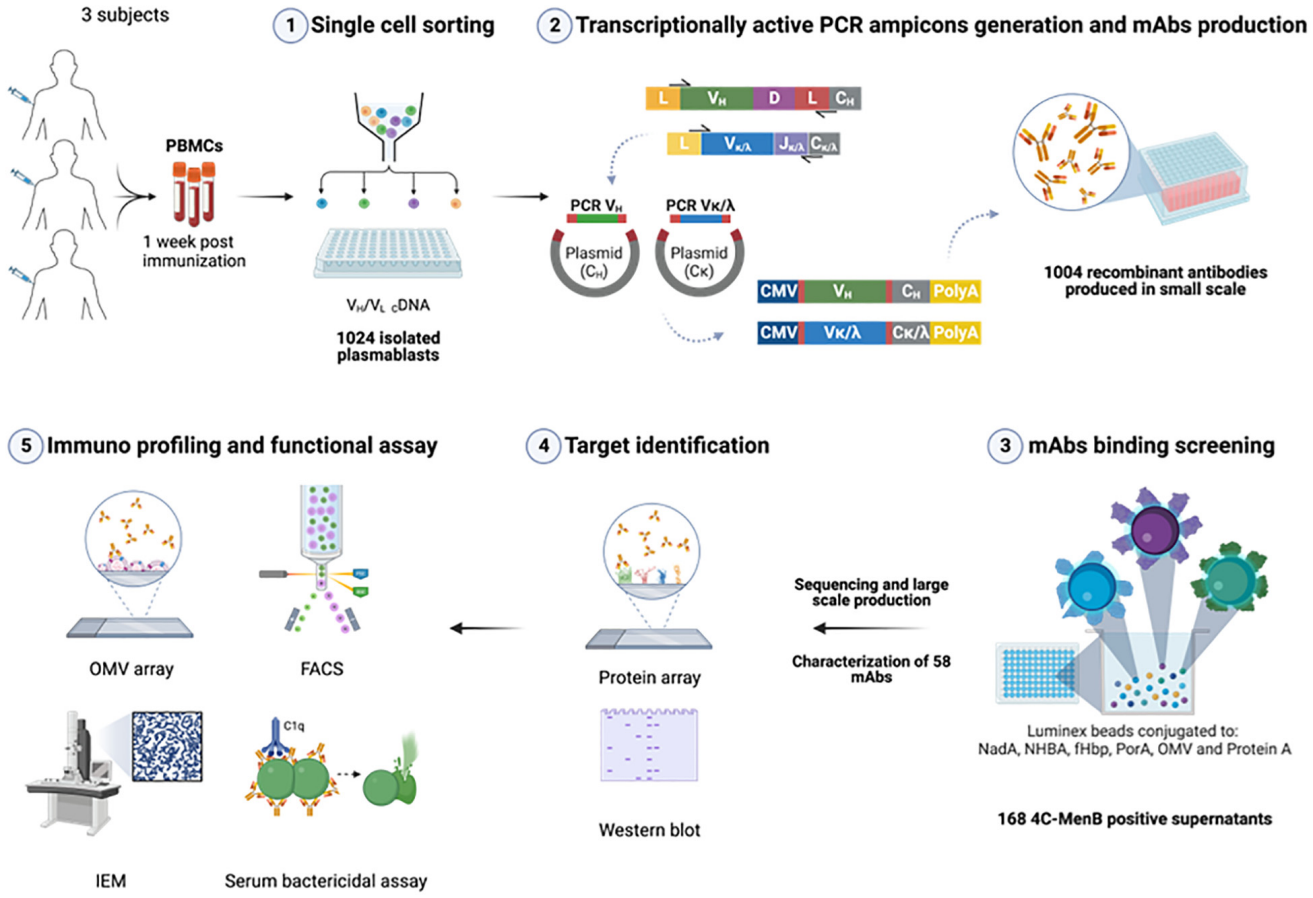
Exploitation of the human antibody repertoire induced by 4CMenB vaccination to elucidate the contribution of OMV component to cross-protection. Plasmablasts (PBs) isolated from blood of selected subjects 1 week after the last immunization were single cell sorted (1), retrotranscribed and IgG variable region of Heavy (VH) and Light (VL) chains were amplified and cloned into IgG1 expression plasmids. Minimal gene fragments to allow recombinant mAbs expression (transcriptionally active PCR amplicons, TAP) were amplified and used to co-transfect mammalian cell line Expi293 for small scale expression in 96w plates (2). Raw supernatants were tested in Luminex assay for IgG quantification and binding specificity of the expressed HumAbs (3). 100 OMV-specific HumAbs were sequenced and unique HumAbs were expressed at larger scale and 58 purified HumAbs were then tested on protein microarray and Western blot for target identification (4). HumAbs were further characterized and assessed for functionality with different approaches (5). Created in BioRender. Cinelli, P. (2025) https://BioRender.com/vbcb0d5.

**Figure 2:**
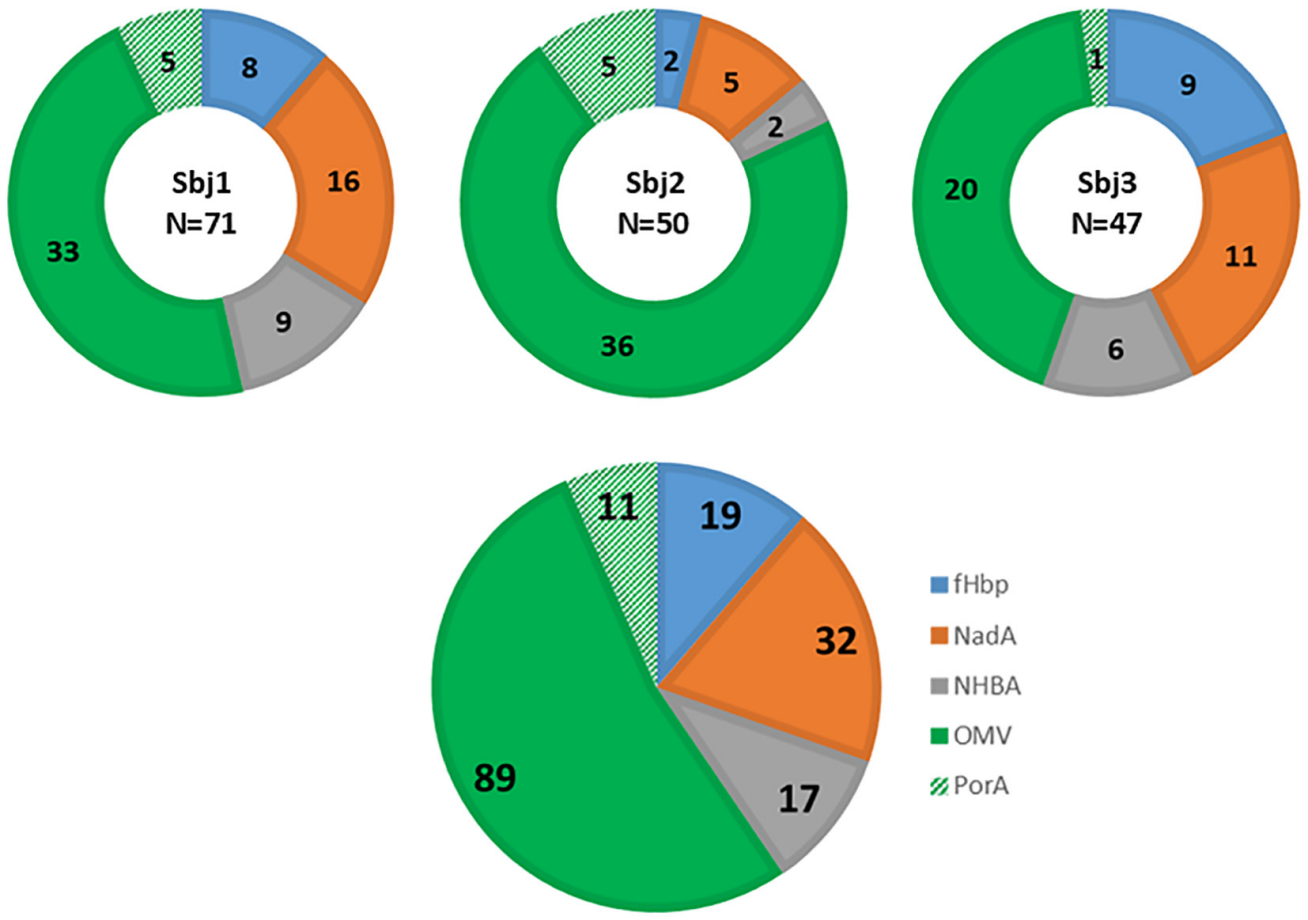
Luminex analysis of binding specificity of TAP-expressed recombinant HumAbs derived from PBs isolated from three subjects immunized with 4CMenB. Representation of specificity distribution of recombinant HumAbs for the 4 main antigen components as well as PorA (NadA, NHBA-GNA1030, GNA2091-fHbp, OMV, and recombinant PorA_P1.7 2.4), highlighted with different colors. Luminex beads were conjugated with each component (GNA2091-fHBP, blue; NHBA-GNA1030, grey; NadA; red, and OMV from NZ98/254, green; and the recombinantly expressed PorA, hashed green). Doughnut pies represent distribution and absolute numbers for each subject (N reported in the center of doughnut pies represents the total number of 4CMenB-specific mAbs identified for the subject). Pie chart represents the overall distribution and absolute numbers of the 168 4CMenB-specific HumAbs identified.

For the identification of 4CMenB-specific PBs and OMV-specific HumAbs we used the approach depicted in **Figure 1**. PBs were stained and sorted by flow cytometry, from PBMCs of each of the three subjects, as positive for CD27, CD38 and CD19 markers and negative for CD20 and IgD receptor and were isolated as single cells. The variable regions of paired heavy and light chains (VH and VL, respectively) were amplified from single PBs and ligated into linearized plasmids containing the constant chain of a human IgG1 and the constant chain of human Igk, respectively. The IgG1 constant chain included an Fc modification (E345R/E430G or E345R/E430G/S440Y, Hexabody) to enhance complement activation capability of mAbs (41) to increase the possibility of identifying functional HumAbs in our screening. In a second step, ligation products of paired VH/VL were further amplified to produce Transcriptionally Active PCR (TAP) fragments which were then used to transiently transfect the mammalian cell line Expi293 for small-scale expression of the recombinant HumAbs. Raw supernatants containing secreted antibodies were tested in two different Luminex binding assays: a mono-plex assay with Protein A beads, to quantify the total IgG content, and a multiplex assay with beads coated with fHbp, NHBA, NadA vaccine antigens, the OMV component and the PorA recombinant protein, to evaluate HumAb specificity for the different 4CMenB components. A total of 1024 PBs were sorted and their corresponding recombinant HumAbs were expressed with a success rate of 98%, as revealed by a concentration of IgG > 1 ng/ml measured in the supernatant of 1004 transfected cells. Among the 1004 IgG-positive supernatants screened, a total of 168, corresponding approximately to 16%, were positive for one of the 4CMenB components as determined by the multiplex 4CMenB component Luminex assay. The distribution of the overall and subject-related HumAb antigen specificity is depicted in **Figure 2**. Overall, 100 out of the 168 HumAbs (representing approximately 60% of 4CMenB-specific antibodies) resulted positive for OMV (**Figure 2**, green) and, quite surprisingly, only 11 of these recognized the recombinant PorA protein, commonly considered as the immunodominant antigen in the OMV (**Figure 2**, dashed green). The specificity of the remaining HumAbs was distributed among the recombinant antigens present in 4CMenB, with a slight prevalence for anti-NadA mAbs (**Figure 2**, orange), in line with previous findings (5).

The entire set of OMV-positive supernatants, including those specific for PorA, was tested for functionality in a bactericidal assay, which measures the ability of the HumAbs to engage the complement on the surface of the bacterium resulting in bacterial lysis. The bactericidal assay was performed with two different meningococcal strains: NZ98/254, the isolate from which the OMV of 4CMenB are produced and which is the reference strain for the vaccine PorA protein (P1.7 - 2.4), and the strain M07576, mismatched for the PorA antigen (P1.22-2.14) and selected because of its high susceptibility to the OMV-mediated killing from human sera (16). These two strains were used to discriminate the PorA contribution to bacterial killing. Out of 100 raw supernatants tested, 29 showed bactericidal activity at a dilution ≥ 2 on the selected strains, including 6 of the 11 PorA-positive HumAb supernatants and 23 of the 89 OMV-specific supernatants (**Supplementary Figure S2**). In general, all the PorA-specific HumAbs with killing ability (Sbj1_mAb1, Sbj1_mAb23, Sbj1_mAb30, Sbj2_mAb9, Sbj2_mAb29 and Sbj3_mAb3) exhibited higher bactericidal activity towards NZ98/254 than M07576. The other non-bactericidal 5 supernatants were estimated at Luminex to have low levels of expressed IgG (**Supplementary Table 2**). On the contrary, all the other functional OMV-specific supernatants surprisingly showed no or low activity against the NZ98/254 strain, with the exception of Sbj1_mAb16, and higher bactericidal activity towards the M07576 strain, in line with the observation that these HumAbs recognize a non-PorA meningococcal antigen. We considered of lower interest HumAbs with high concentration in the supernatant (>10 ng/mL), low signal in the Luminex binding assay (<1000 MFI) and no bactericidal activity as raw supernatant. Based on this assumption, we prioritized a subset of 66 HumAbs for large-scale expression and purification, based on their positive activity in the bactericidal assay in supernatants and/or their relative binding signal on the vaccine OMV in the Luminex assay.

### Specificity of 4CMenB-induced HumAbs revealed that PorB is a highly immunogenic OMV antigen able to induce bactericidal activity

**Figure 3:**
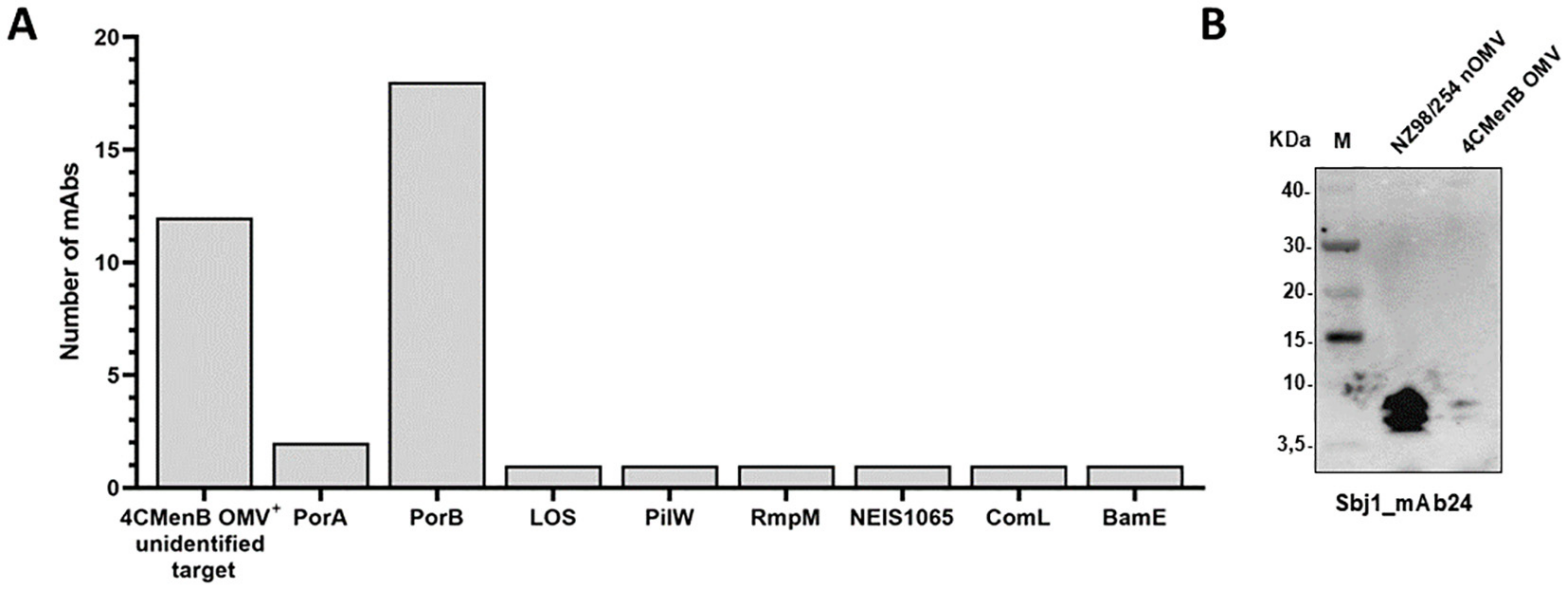
Identification of HumAbs cognate target by protein microarray and Western blot. **(A)** Representation of the targets identified by protein microarray and western blot analysis for 38 of the 58 tested HumAbs. Each bar represents the number of antibodies reactive for the specific antigen reported in the horizontal axis (numbers reported in **(A)** are derived from data described in **Supplementary Figure S3**). **(B)** Western blot analysis of the only LOS-specific identified HumAb, Sbj1_mAb24. The analysis has been performed on extracts from NZ98/254 nOMV and 4CMenB OMV, numbers on the left report the molecular weight of the Novex sharp pre-stained protein standard (M) represented in the first lane of the gel.

To further investigate the specificity and functionality of the selected HumAbs, we cloned codon optimized gene fragments into expression vectors and attempted their production at larger scale. We successfully obtained 58 HumAbs in the recombinant form, including two out of the eight PorA-specific HumAbs. The specificity of the purified HumAbs was determined using a protein microarray previously described (16), encompassing approximately 30 of the most abundant proteins found in OMV vaccine lots. All the purified HumAbs were tested at a normalized concentration and results are summarized in **Figure 3A** and **Supplementary Figure 3**. A total of 26 out of the 58 HumAbs tested were able to recognize at least one antigen present on the protein microarray. In particular, the most recognized antigen was PorB, for which 18 antibodies resulted specific. The 2 HumAbs previously defined as anti-PorA were confirmed by microarray analysis able to recognize the PorA antigen either as recombinant or when expressed in GMMA. Finally, the antigens RmpM (NEIS1783), BamE (NEIS0196), PilW (NEIS1264), ComL (NEIS0653) and the hypothetical protein NEIS1065 were recognized by one single HumAb each, namely Sbj3_mAb16, Sbj3_mAb9, Sbj1_mAb22, Sbj3_mAb6 and Sbj3_mAb4 respectively (**Figure 3A**, **Supplementary Figure 3**). Interestingly, 12 HumAbs reacted positively only with the NZ98/254 OMV suggesting that the target antigen was not represented in the protein microarray, preventing its identification. Surprisingly, 22 of the tested HumAbs, almost 1/3, did not react with any specific antigen nor with the NZ98/254 OMV on the array, conflicting with the Luminex results (**Supplementary Figure 2**). We hypothesized, given the higher sensitivity of Luminex technique with respect to the protein array, that these 20 HumAbs might have a target with very low abundance in the OMV bacterial membrane and we did not investigate them any further.

When multiple positive signals were obtained for the same HumAb according to protein microarray screening, the specific target was confirmed by Western blot analysis performed with specific recombinant proteins, vaccine OMV (detergent extracted) and/or OMV naturally released in culture supernatants during bacteria growth and hereafter called nOMV (native outer membrane vesicles) (**Supplementary Figure 3B**). Interestingly, when the Sbj1_mAb24 was probed by Western blot it reacted with two low molecular weight bands in both OMV preparations (**Figure 3B**). This profile was compatible with the recognition of the meningococcal lipooligosaccharide (LOS), that normally run in SDS-page with apparent molecular weight between 3.5 and 10 kDa. In line with this, the bands were clearly visible in the nOMV preparation and barely detectable in the OMV sample, in which the LOS is largely lost due to the detergent extraction of the preparation.

In conclusion, the protein microarray and Western blot screening were instrumental in discovering the targets of 26 HumAbs identifying highly immunogenic antigens of the OMV able to trigger potentially protective immune responses.

### PorB- and LOS-specific mAbs show bactericidal activity against a panel of MenB strains

In order to investigate the functional activity of 36 cloned recombinant antibodies, including the 12 OMV+ mAbs with unknown targets and the 24 mAbs with known non-PorA targets, a panel of 18 different meningococcal strains were selected (**Supplementary Table 3**). The 18 MenB strains have been previously shown to be susceptible to killing mediated by sera from 4CMenB vaccinees but the bactericidal activity was largely not expected to be mediated by the major antigenic vaccine components (fHbp, NHBA, NadA and PorA) as these strains are largely mismatched in the strain panel. In particular, 7 of the 18 strains (namely M07 0241084, M07576, M09929, M08389, M14569, M12898, and LNP24651) had previously shown susceptibility in SBA to sera from infants vaccinated with 4CMenB formulation but not with the rMenB formulation (fHbp, NHBA, and NadA only) and were recently reported to be suitable OMV-indicator strains (16). They include the PorB reference strains M07576 and M09929. Five strains from Argentina that were MATS negative but showed susceptibility to 4CMenB infant sera were included (50). In addition, NZ98/254 as PorA indicator strain and M13520 and M07463, M13547, M08129 and M18717 were included as they resulted susceptible in the serum bactericidal assay performed with sera from 4CMenB vaccinated subjects.

**Figure 4:**
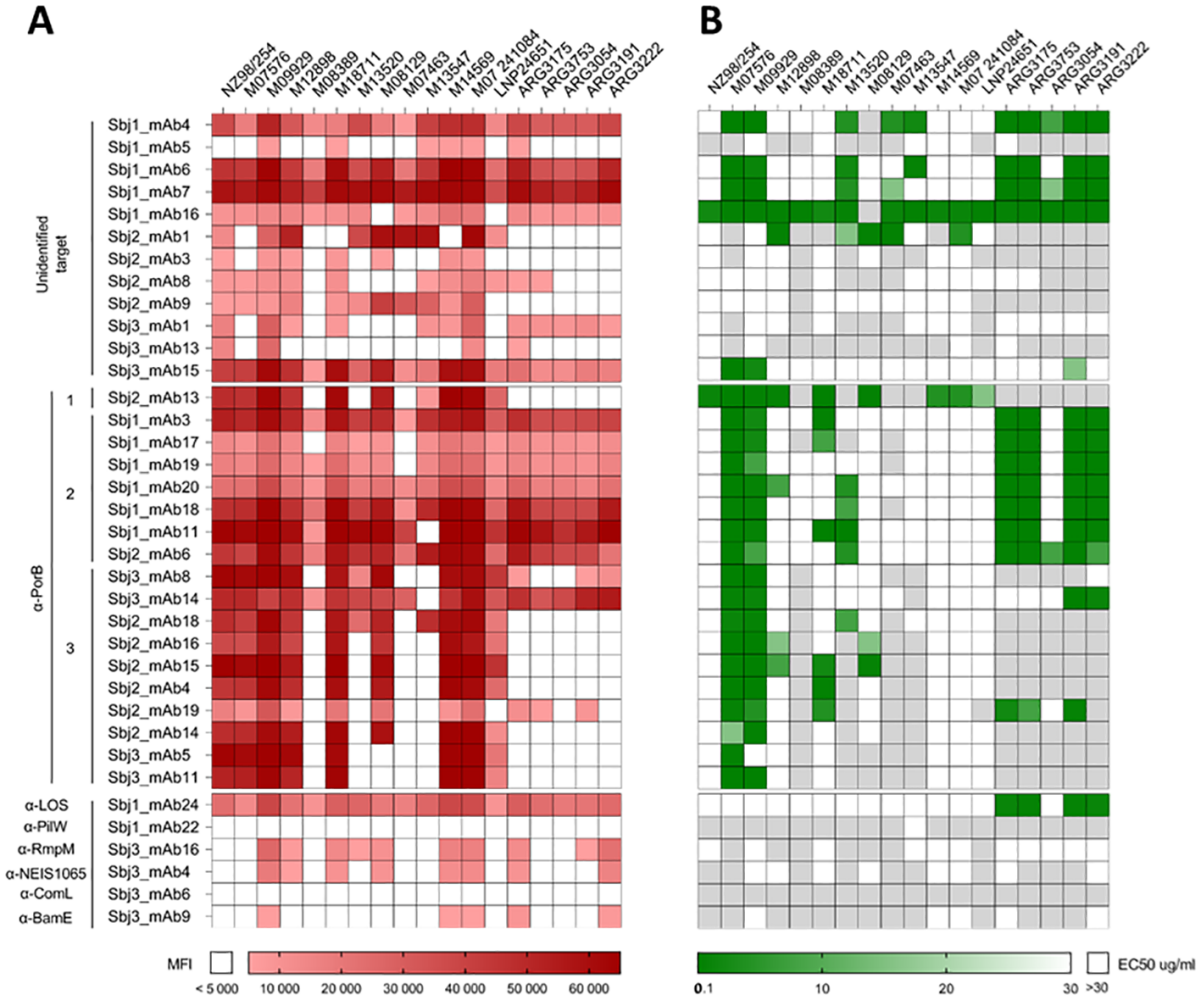
Binding and functional characterization of HumAbs on a panel of nOMV purified from 18 MenB strains. **(A)** Heatmap representing the reactivity (Mean Fluorescence Intensity, MFI) of the 36 purified HumAbs (12 OMV-specific with unknown target, 18 PorB-specific and 6 mAbs directed against OMV subdominant antigens) against native OMVs purified from the supernatant of 18 selected MenB strains. White boxes represent values below cut-off of positivity (MFI < 5000). **(B)** Heatmap representing the EC50 of each HumAb against the tested strain. White boxes indicate absence of bactericidal activity at the highest mAb concentration tested (30µg/ml) while light grey boxes indicate that mAbs were not tested on the relative strain.

In order to test the reactivity of the 36 HumAbs to surface antigens across the entire panel of meningococcal isolates, we generated a multi-strain OMV microarray from the panel for high throughput simultaneous binding analysis with low amount of HumAb. Native OMV (nOMV) were purified from supernatants after bacterial growth and, following quality assessment, nOMV were spotted onto nitrocellulose-coated glass slides along with the NZ98/254 OMV component from 4CMenB as positive control. All the HumAbs were then tested for binding on this multi-strain microarray and their reactivity was assessed as mean fluorescence intensity (MFI) values on each OMV sample. Hybridization results are summarized in the heatmap of **Figure 4A** where almost all the tested mAbs showed a signal higher than the cut-off on at least one of the 18 nOMVs spotted on the array, exhibiting strain-specific binding profiles. Around 50% of HumAbs were highly cross-reactive, showing binding on most of the tested strains, even if with different intensities. Only the PilW- and ComL-specific mAb did not react with any OMV on the array.

Based on the results of the OMV array, the 36 mAbs with positive signals were tested for functionality in a bactericidal assay in presence of a rabbit complement source on the strains for which they showed recognition (**Figure 4B**). Six of the 12 mAbs with unidentified target showed no bactericidal activity on any of the tested strains, while the remaining 6 were able to kill at least 3 strains. The highest bactericidal activity across the panel of strains was observed with Sbj1_mAb16, exhibiting cross-killing on all of the 17 tested strains. The 5 HumAbs with known protein target (i.e. PilW, RmpM, BamE, ComL, and NEIS1065) did not show any bactericidal activity on the tested strains, while the LOS-specific HumAb Sbj1_mAb24 was able to mediate bactericidal killing on four of the five Argentinian strains tested. Interestingly, our analysis revealed that all the anti-PorB mAbs were able to kill at least one of the MenB strains tested, with the majority of HumAbs (12 out of 18) being cross-functional on more than 3 strains. Hierarchical clustering of the PorB-specific mAbs based on their binding and killing features revealed three major groups, as shown in **Figure 4**. Cluster 1, constituted by a single HumAb (Sbj2_mAb13) was able to kill all the MenB strains recognized on the OMV array. Cluster 2 included 6 HumAbs from Sbj1 and 1 HumAb from Sbj2, and while these mAbs recognized the majority of the strains on the OMV array they were able to kill only a subgroup of them which included most of the Argentinian strains tested. Cluster 3, composed of 10 HumAbs from subjects 2 and 3, whose mAbs were able to recognize a reduced number of strains on the array and to kill mainly the PorB reference strains M07576 and M09929. Overall, we observed that PorB HumAbs with similar cross-binding and cross-killing behaviors isolated from different subjects could be grouped by these features.

From this analysis, the correlation between OMV binding and the bactericidal activity on the corresponding strain of our HumAbs existed only for cluster 1 mAb, Sbj2_mAb13. The strains M07576 and M09929, that are considered reference strains for PorB-mediated killing were indeed recognized and killed by almost all the PorB-specific mAbs tested (except for Sbj3_mAb5, that did not show functional activity on M09929). However, we identified strains that were recognized by almost all HumAbs on the OMV array (such as M14569, M07 241084 and LNP24651) but were only killed by one HumAb in bactericidal assay. On the other hand, we identified some highly cross reactive mAbs (such as the mAbs in Cluster 2 and the Sbj3_mAb14 in Cluster 3) that were able to kill only a subset of the recognized strains. This lack of consistency between binding to the OMV and killing of the corresponding strain for the PorB-specific mAbs could be ascribed to a low accessibility or density of the mAbs to PorB on the bacterial surface, suggesting that PorB *per se* might be accessible on the bacterial surface but in an epitope-dependent manner.

### Characterization of PorB-specific mAbs binding on meningococcal strains

**Figure 5:**
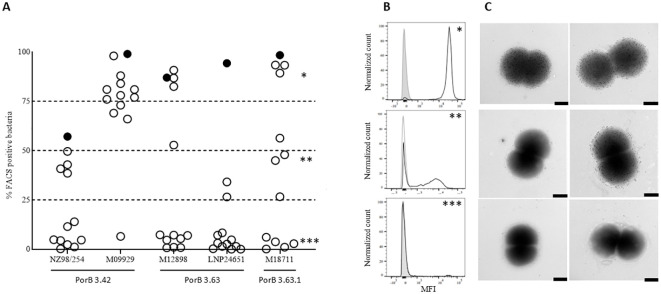
Accessibility of HumAbs epitopes on different MenB strains: **(A)** FACS analysis of 13 PorB-specific HumAbs binding to the surface of 5 MenB strains. On the X axis are reported the 5 different MenB strains and the PorB variant expressed. On the Y axis is reported, for each strain, the percentage of bacteria showing a fluorescence intensity superior to the secondary mAb alone when incubated with the different strains. Full circle represents the only HumAb belonging to Cluster 1 (Sbj2_mAb13). **(B)** Histograms showing the binding profile of 3 representative HumAbs on the MenB strain M18711 and identified by asterisks in panel A: the upper panel (*) shows the binding profile of a HumAb binding the surface of the whole bacterial population (Sbj2_mAb4); the central panel (**) shows the binding profile of a HumAb able to bind the surface of almost 50% of the bacterial population with good intensity, while the remaining 50% of the population is almost completely negative (Sbj3_mAb5); the lower panel (***) shows the binding profile of a HumAb not binding the selected strain (Sbj1_mAb19). **(C)** Immunogold analysis of the binding profile of 3 representative HumAbs on the MenB strain M18711: the upper panels shows the binding profile of a HumAb binding the surface of the whole bacterial population; the central panel shows the binding profile of a HumAb able to bind the surface of almost 50% of the bacterial population with good intensity, while the remaining 50% of the population is almost completely negative; the lower panel shows the binding profile of a HumAb not binding the selected strain. Two representative images are reported for each HumAb, black bar represents 100nm.

To further characterize the accessibility of the PorB protein on the bacterial surface of different meningococcal strains, we selected a subset of 13 PorB-specific mAbs, representative of the different behaviors observed, and analyzed 5 strains with different susceptibility to PorB-mediated killing by FACS surface staining experiments. Bacteria were collected in early exponential phase and incubated with the monoclonal antibodies. The binding of the mAbs to the different strains was then revealed using fluorescently labelled secondary antibodies. Secondary antibody alone, as well as an unrelated mAbs, were used as negative controls. As shown in **Figure 5A**, FACS experiments revealed not only that the accessibility of PorB is different among different strains, as we might expect, but also that the bacterial population in the same preparation is not homogeneous for the accessibility of this antigen. The strain M09929 was recently shown to be sensitive to PorB-dependent killing and, in line with this finding, all the mAbs but one (Sbj3_mAb5, not bactericidal on this strain) were able to bind the surface of this strain. On the contrary, the NZ98/254 strain, that shared an identical PorB (variant 3.42), was negative for the majority of the tested mAbs, in line with the already described low accessibility of PorB on this strain (16). The only HumAb tested able to bind more than 50% of the bacterial population for this strain was Sbj2_mAb13 (cluster 1), that resulted also the only one bactericidal on this strain. Interestingly, another 4 weakly FACS-positive mAbs on NZ98/254 were able to recognize less than 50% of bacteria after staining. The LNP24651 strain, that carried a different PorB variant (PorB 3.63) and was a strain resistant to PorB-mediated bactericidal killing with the mAbs identified in this study, was also poorly recognized by the HumAbs except for Sbj2_mAb13 (Cluster 1) which kills this strain. As for the strains M12898 (bearing a similar PorB3.63 to LNP24651), and M18711 (bearing a different PorB3 variant) the binding pattern observed in FACS with the panel of mAbs was more heterogeneous: we observed the three representative binding patterns, 1) mAbs able to bind the entire bacterial population (*), 2) mAbs able to bind only a sub-population of bacteria, around 50% or less (**), and 3) mAbs that were largely negative (***) (**Figure 5A**). **Figure 5B** shows the MFI per count FACS scan for 3 mAbs with representative binding patterns on the strain M18711, highlighting the diverse FACS-positive subpopulation patterns. This different binding behavior of HumAbs was confirmed by immunoelectron microscopy (IEM) analysis (**Figure 5C**), in which we clearly see a mixed population of gold-particle coated or negative bacteria for the weakly FACS positive mAb with intermediate binding around 50% or less (**). This is indicative of a bi-phasic behavior in which bacterial subpopulations within the same strain exhibit binding or no-binding and interestingly this bi-phasic behavior is measured only for the cluster 2 and cluster 3 mAbs, and not for cluster 1 which binds highly and kills all strains tested. We interpret this biphasic behavior of the PorB mAbs to suggest that the accessibility of PorB epitopes on the surface of different meningococcal strains could be masked by other membrane components that seem subjected to phase variation.

### VH4 and VH3 gene families dominate the PorB-specific mAbs repertoires

**Figure 6:**
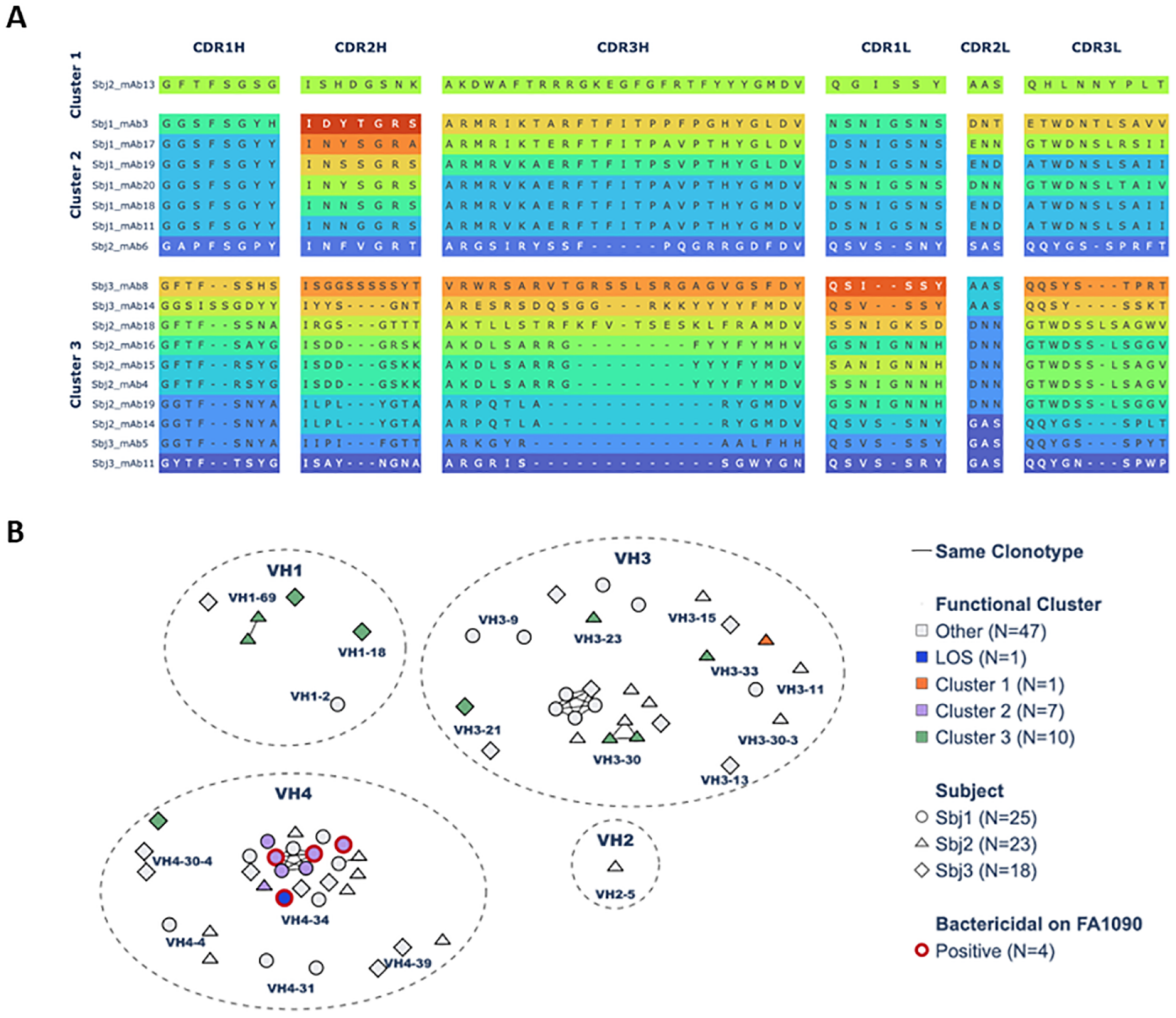
Sequence analysis of PorB-specific HumAbs: **(A)** Heavy and Light chain CDRs sequence alignment of PorB-specific HumAbs. Sequences are grouped according to the 3 identified functional clusters of the HumAbs and vertically ordered, inside the same cluster, according to length and similarity of the Heavy chain CDR3 (CDR3H). Different colors within each box differentiate CDRs with at least one mutation at residue level. Gap positions (-) are inserted according to ANARCI alignment at cluster level following IMGT numbering scheme. **(B)** Clonotypes network of HumAbs identified in this study in a 2D map representing Heavy chain sequences properties. HumAbs, each represented by an individual symbol, are closer in the map if they share the V family, V gene and J gene, as well as if they have similar CDR3 sequence. Symbols connected by a solid black line are assigned to the same clonotype. Groups of mAbs using the same Heavy chain V gene family are surrounded by a dashed circle reporting the corresponding family label. Orange, lilac and green color of each symbol indicates the functional cluster of the PorB-specific mAbs while blue identifies the LOS-specific mAb and grey identifies mAbs with other specificity. The shape indicates the subject from which each mAb has been isolated while the red edge line identifies mAbs resulted bactericidal against Gonococcus FA1090 strain.

We analyzed the PorB mAbs of the three clusters identified by functional data clustering in terms of gene usage and sequence heterogeneity (**Supplementary Table 5**). To shed light on their sequence characteristics, we performed multiple sequence alignments of CDR3 regions (**Figure 6A**) and clonotype analysis of mutations and the variety of V-J genes pairing were sufficient to segregate the sequences into multiple clonotypes (**Figure 6B**). The 10 mAbs in cluster 3 showed high diversity both in terms of CDR3H aminoacidic sequence length and identity (**Figure 6A**) as well as diversity in VH gene usage (**Figure 6B**) since sequences are spread across VH1, VH3 and VH4 gene families. Three mAbs shared the same VH1-69 gene, one mAb used a different VH1 family gene (VH1-18) while the rest of members mainly used different VH3 family genes and one of VH4 family (VH4-30-4). Conversely, cluster 2 mAbs showed highly polarized VH usage and were all rearranged with the VH4-34 germline, often pairing with same light chain V gene (VL1-51) (**Supplementary Table 5**). Furthermore cluster 2 exhibit high CDR3H sequence similarity (**Figure 6A**). This may be related back to the origin of the monoclonals, with 6 out of the 7 sequences coming from the same donor Sbj1 and being identified by clonotype analysis as one large clonal family within the VH4-34 germline (**Figure 6B**).

### *In silico* docking of PorB-specific HumAbs on NZ98/254 PorB model reveals different loops preferentially bound by the 3 clusters

**Figure 7:**
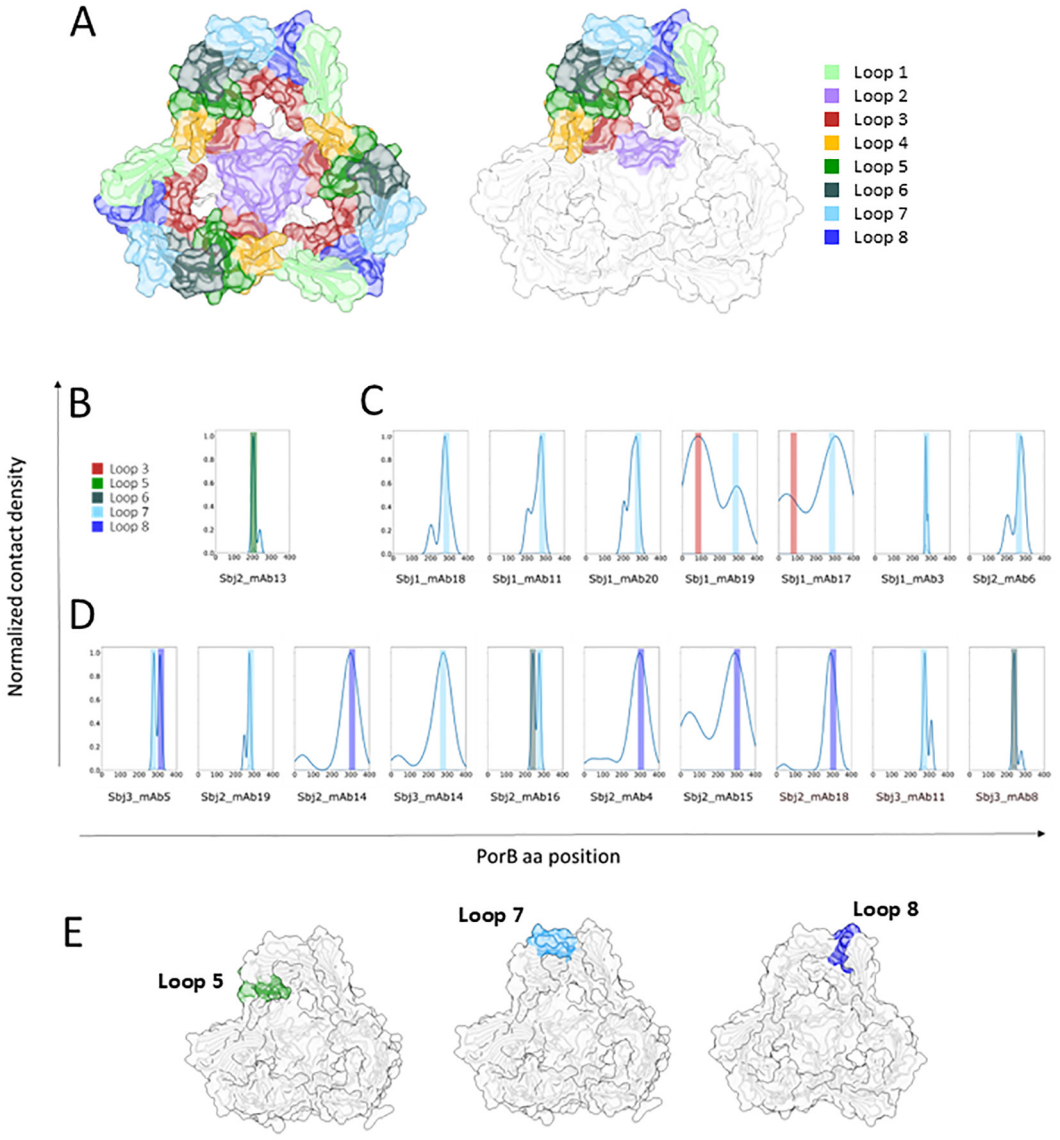
Representation of AF model of trimeric PorB NZ98/254 and in silico binding profile analysis of anti PorB HumAbs: **(A)** AF2 multimer model of PorB forming a trimer. The whole model is represented in white cartoon and surface while the 8 loops facing outwards the OM are highlighted with different colors on all the three monomers (left) or only on one monomer (right) to highlight the trimeric assembly of PorB. Loops are colored as follows: loop1 in light green, loop2 in medium purple, loop3 in brown, loop4 in orange, loop5 in green, loop6 in dark slate gray, loop7 in light blue and loop8 in dark blue. Binding profile analysis conducted on the best docking pose of each HumAb (reported below in the histogram) for each of the PorB HumAbs functional clusters: **(B)** cluster 1, **(C)** cluster 2, and **(D)** cluster 3. Histograms represent the relative number of poses (Y axis) for the individual HumAbs on each aminoacidic position (X axis), color bars highlight the interacting loop. **(E)** Structural models with the loops preferentially interacting with each HumAb cluster highlighted (loop5 for cluster 1 on the left, loop7 for cluster 2 in the middle and loop 8 for cluster 3 on the right).

To better understand the possible implications, in terms of physico-chemical and structural properties, of the observed mutations, we decided to further investigate them through computational structure modeling and docking experiments. Given the results described before, we were interested in better defining the epitopes recognized by the diverse clusters of mAbs on PorB. With this aim, the 18 PorB-specific mAbs were further analyzed with an *in-silico* docking approach to the immunogen PorB protein of the NZ98/254 strain. The foundation for each docking analysis requires the molecular structures and as diverse PorB allele structures have been solved and characterized as trimeric (51–53), we employed AlphaFold2 Multimer to computationally predict a trimeric conformation of the NZ98/254 PorB allele. As shown in **Figure 7A**, the resulting model is a trimeric complex with 6 out of 8 loops located in the outward facing portion while loop2 and loop3 remain inside the ß-barrel, with the first located at the interface between the monomers. A total of eight regions were identified as potential binding sites, defined by the following aminoacidic positions: 39-46 (loop1), 81-95 (loop2), 113-143 (loop3), 164-176 (loop4), 197-210 (loop5), 234-249 (loop6), 270-284 (loop7) and 309-319 (loop8) (**Figure 7A**). For the structural prediction of the paratope region from each of the HumAb sequences, DeepAb, an AI algorithm specific for antibody modeling that provides a highly confident estimation of the CDR3 region, was used while the paratope region was predicted with Paragraph (54) on the generated models. Each of the 18 PorB mAbs paratope structures were docked to the modeled trimeric PorB with multiple iterations of the HADDOCK software, and the docking interface for each HumAb/PorB pair was identified as the functional cluster giving rise to the lowest energy pose and therefore the most representative antigen/antibody interaction. **Figures 7B–D** shows the docking analysis with individual histograms for the highest relative number of poses (Y axis) on each aminoacidic position of PorB (X axis) for the each HumAb from cluster 1 (**Figure 7B**), cluster 2 (**Figure 7C**), and cluster 3 (**Figure 7D**), highlighting the loops with which they interact and the likely epitopes for each. The Sbj2_mAb13 HumAb (cluster 1) showed a preferential binding on the NZ98/254 PorB loop5 (**Figure 7B**) and the epitope is highlighted in **Figure 7E** (left panel). Binding profile analysis conducted on the best docking pose from mAbs from cluster 2, composed of mAbs that showed high sequence similarity for both heavy and light chain CDRs (**Figure 6A**), revealed a preferential interaction with the PorB loop7, that was bound by all HumAbs mostly as single loop (5/7 tested mAbs) or in combination with PorB loop3 (2/7 tested mAbs) (**Figures 7C, E**, central panel). The third cluster (Cluster 3) includes HumAbs exhibiting high sequence heterogeneity for both heavy and light chain CDRs (**Figure 6A**), and, not surprisingly, in silico docking revealed a mixed PorB binding profile (**Figure 7D**): the most representative group of mAbs (5/10) interacted with PorB loop8 alone or in combination with loop7; another group of mAbs (4/10) that interact with PorB loop7 alone or in interaction with loop6; and one that interacts with loop6 only. While the docking highlights heterogeneity of the epitopes from this cluster, in line with the heterogeneity of the functional responses observed, the most representative epitope from this cluster, loop 8, is highlighted in **Figure 7E** (right panel).

### PorB and LOS HumAbs are able to bind multiple gonococcal strains and are bactericidal against *Neisseriae gonorrhoeae* FA1090 strain

**Figure 8:**
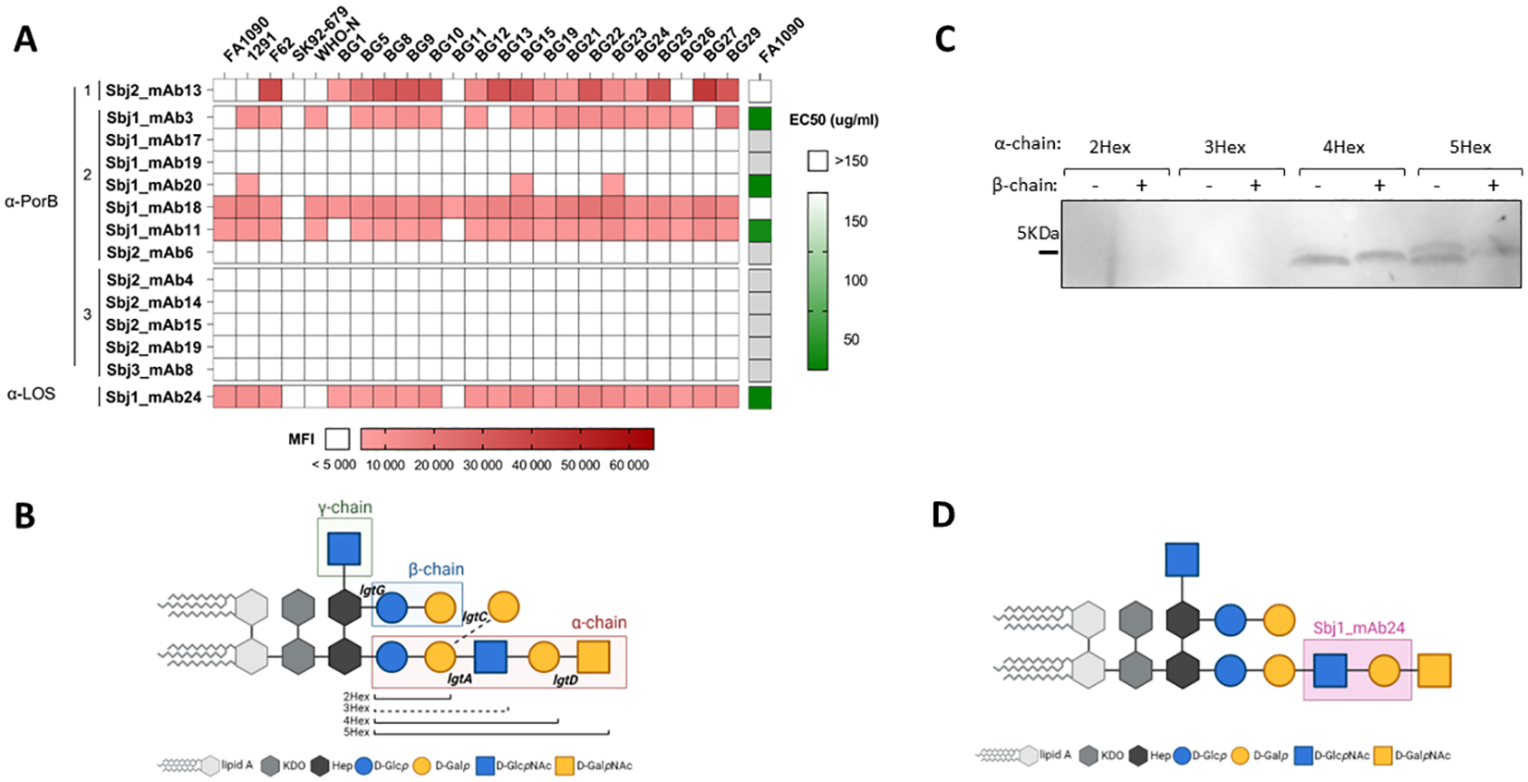
Characterization of HumAbs on *N.gonorrhoeae*: **(A)** Binding and functionality of PorB- and LOS-specific mAbs on gonococcus: red heatmap on the left represents the reactivity (MFI) of the 14 HumAbs (13 PorB specific and 1 mAb directed against LOS) against OMV from the 23 selected gonococcal strains. White boxes represent values below cut-off of positivity (MFI < 5000). Green heatmap on the right shows the EC50 of each mAb against FA1090 gonococcal strain. White boxes represent EC50 higher than the cut-off of 150 µg/ml, while light grey boxes indicate mAbs not tested in SBA. **(B)** Schematic representation of gonococcal LOS structures, with the 3 chains boxed with different colors and the relevant *lgt* genes involved in its biosynthesis reported in the respective attachment sites. The alternative α-chain, through activity of the *lgtC* gene, is highlighted by dotted line and nominated the 3Hex structure. **(C)** Binding profile of LOS-specific mAb Sbj1_mAb24 on a panel of 8 different gonococcal mutant strains on the MS11 genetic background, each one exposing largely a single LOS structures reported in the legend. **(D)** Identification of the putative epitope recognized by the LOS-specific mAb Sbj1_mAb24 based on the Western blot results. Created in BioRender. Cinelli, P. (2025) https://BioRender.com/9k43x2j.

Our results suggest that HumAbs elicited by 4CMenB against PorB and LOS may enhance the breadth of coverage of this vaccine on meningococcal strains. To investigate whether HumAbs identified in our study against these antigens may be involved in the cross-protection against Gonococcus induced by 4CMenB vaccination in humans, we investigated binding and functional activity of the HumAbs against gonococcal strains. An OMV array printed with native OMVs purified from 23 different gonococcal strains, representative of both laboratory and circulating strains, was used to screen gonococcal surface antigen binding of a subset of 13 PorB-specific mAbs belonging to all the 3 clusters, together with the only LOS-specific mAb identified in this study. Five PorB mAbs belonging to clusters 1 and 2, and the LOS-specific HumAb, were able to recognize OMV derived from the selected *N.gonorrhoeae* strains (**Figure 8A**, red heatmap), with different binding profiles. Interestingly, cluster 1 did not show binding to OMV from the PorB1A strains in the panel (SK92-679, WHO-N and BG11), while some cluster 2 mAbs bound both PorB1B and PorB1A, and cluster 3 did not bind gonococcus OMV at all. We tested the ability of the 6 mAbs to kill the gonococcal strain FA1090 through a bactericidal assay performed with human serum as complement source. Three PorB-specific mAbs (from cluster 2) and the LOS-specific mAb showed bactericidal activity on FA1090 (**Figure 8A**, green heatmap), suggesting that PorB and LOS antibodies may cross-bind and kill gonococcal strains with cross-recognized epitopes adequately presented on their surface.

### Identification of the LOS epitope bound by the 4CMenB-elicited by the Sbj1_mAb24 cross-functional mAb

LOS from meningococcus and gonococcus share partial similarity (55, 56) and consist of a lipid An anchor connected by a heptose (Hep) and KDO containing inner core to the outer oligosaccharide extensions. The biosynthesis of LOS in *Neisseria* spp is under the control of phase variable *lgt* genes (57) and can be synthesized with diverse oligosaccharide structures, depending on the complement of LOS biosynthetic genes expressed in each single bacterium (**Figure 8B**). While the γ-chain is constant, the *lgtG* phase-variable (PV) gene controls the β-chain glycan extension from the Hep2 core heptose and the PV *lgtA*, *lgtC* and *lgtD* genes control α-chain glycan extensions from the Hep1 core heptose and depending on which *lgt* genes are expressed may be of variable length or consist of an alternate α-chain structure. As a result of PV *lgt* genes LOS structures expressed by *Neisserial* strains may vary considerably. In order to define the putative LOS epitope recognized by the specific Sbj1_mAb24 antibody, we performed Western blot analysis on 8 different MS11 gonococcal strains genetically engineered to express only one LOS structure (58). In each MS11 mutant strain, the phase variable *lgt* loci (*lgtA, C, D* and *G*) were genetically fixed either ‘ON’ or ‘OFF’ in different combinations giving rise to 8 possible glycan structures characterized by different number of hexose (Hex) sugars in the α-chain (2Hex, 3Hex, 4Hex and 5Hex), with or without the β-chain (β+ or β-, respectively) as represented in **Figure 8B**. Western blot analysis showed that the Sbj1_mAb24 HumAb was able to recognize LOS structures characterized by 4 or 5 hexoses in the α-chain (4Hex and 5Hex), regardless of the presence of the β-chain, while it did not cross-react with the shorter 2Hex and 3Hex structures (**Figure 8C**). This binding profile was consistent with the LOS structure present on the 4CMenB OMV component as the NZ98/254 MenB strain expresses mainly L1 and L3,7,9 LOS, the latter of which consists of the extended α-chain (7). We conclude that the specific epitope recognized by the Sbj1_mAb24 HumAb is likely contained in the extended α-chain mAb between the 2Hex and 4Hex structures and consisting of the GlcNAc–Gal sugar moiety, as highlighted in **Figure 8D**.

## Discussion

Real world evidence from different countries have demonstrated that OMV-based vaccines can provide broader than expected protection against meningococcal disease (27) and moderate protection against gonorrhoeae (59). To assess the breadth of coverage of the OMV-based MenB vaccines, the immunogenicity profile of different OMV formulations have been analyzed and many OMV components other than PorA have been previously identified as immunogenic (16, 60, 61). Recent studies have focused on identifying the contribution of the OMV component of 4CMenB to the full extent of protection against different *N.meningitidis* strains (13, 16) as well as cross-reacting proteins in gonococcal strains (38). In this study, we isolated 36 OMV-specific HumAbs from sorted plasmablasts of three 4CMenB vaccinees in order to identify the target OMV antigens of antibodies that may be responsible for cross protection in humans. The dissection of their target specificity allowed us to identify, in addition to PorA, seven OMV immunogenic antigens, and among those already known - PorB, RmpM, BamE and LOS - we identified the novel immunogenic antigens NEIS1065, PilW and ComL proteins. Interestingly, while the target antigens of one third of the mAbs remain still unidentified, half of the characterized antibodies resulted specific to the Porin B, confirming that this antigen elicits strong responses in humans, unsurprisingly as it is the most abundant OMV antigen estimated around 40% of the total protein, as opposed to 25-30% measured for PorA of the OMV protein content (62). All 18 PorB HumAbs were bactericidal for at least one MenB strain in our OMV-indicator strain panel and, in addition, a single LOS HumAb was also identified as bactericidal. Therefore, through B cell cloning of HumAbs from plasmablasts collected 1 week after 4CMenB vaccination we identified PorB and LOS as antigens in the OMV that elicit functional bactericidal responses and likely contribute to cross protection across MenB strains. The identification of PorB as a bactericidal antigen of the OMV is in agreement with recent work by Viviani and co-authors, where both PorB and OpcA were identified as antigens eliciting responses contributing to the 4CMenB cross-coverage in humans, while NspA was able to mediate bactericidal killing in immunized mice. We did not however identify any HumAb specific for OpcA or NspA. The identification of 6 bactericidal HumAbs with unknown targets suggests that there may be other minor antigens contributing to 4CMenB protective responses in humans and in particular the highly cross-reactive Sbj1_mAb16 HumAb appears very interesting as it targets a common antigen across diverse MenB strains. However, we cannot exclude that these mAbs may recognize conformational epitopes that are not faithfully maintained in recombinantly produced proteins of the microarray or Western blot and these will be the focus of future studies.

The lack of functionality of the RmpM, BamE, NEIS1065, PilW or ComL specific mAbs, despite complement activation mutations included in the Fc portion of the recombinant antibodies, suggests that, although these OMV antigens are immunogenic in humans, their expression levels on bacterial surface may be insufficient to trigger, at least alone, the complement cascade leading to bacteriolysis. However, these antigens may contribute to cooperativity or synergy occurring in polyclonal responses following 4CMenB vaccination (13). We know indeed that the combination of multiple HumAbs, targeting the distinct fHbp and NHBA antigens, while not bactericidal alone, act synergistically in the killing when combined (5, 63).

Through hierarchical clustering of binding and functional behavior and *in silico* docking experiments of the 18 PorB HumAbs, we show that PorB in the OMV may have multiple immunodominant epitopes driving distinct antibody features. *In silico* docking analysis of the binding profile of each mAb suggested loop5, loop7 and loop8 on PorB as the prevalent targets of mAbs belonging to cluster 1, 2 and 3, respectively. Only the unique HumAb belonging to cluster 1 (Sbj2_mAb13) showed bactericidal activity for each of the strains to which it bound suggesting that its predicted epitope, loop5, is highly accessible on the bacterial surface of these strains. Conversely, FACS and EM data showed that the accessibility of epitopes bound by HumAbs belonging to clusters 2 and 3 was variable among different strains, even bearing the same allele of PorB, but also within the same bacterial population. These PorB HumAbs demonstrated biphasic behavior, either binding or not binding to distinct subpopulations within each strain, and as PorB itself is not phase variable the data suggest that loop7 and loop8 accessibility may be masked by another membrane component subject to phase-variation. This different accessibility of PorB on distinct strains has been reported previously (16, 64) and the lack of epitope exposure has been hypothesized to be a shielding effect of the carbohydrate chains of LOS possibly combined with short extra-cellular loops in the PorB protein. Our data demonstrate that the shielding is phase variable and as such may be dependent on phase variable nature of LOS or indeed decorations such as phosphoethanolamine which is also known to be under phase variation (65). Importantly, different loops showed different accessibility in our experiments and the unique HumAb Sbj2_mAb13 from cluster 1 does not appear to exhibit this behavior, suggesting that not all epitopes of PorB may be susceptible to phase variable masking.

Interestingly, all the PorB-specific HumAbs belonging to cluster 2 and two of those belonging to cluster 3 were able to recognize and kill the Argentinian strains tested in this study, together with the LOS-specific mAb, suggesting that multiple non-PorA components of the OMV of 4CMenB contribute together to the cross-coverage of the vaccine on these strains. These strains belong to the ST-865 complex, which was reported by Efron and co-authors as susceptible to 4CMenB-induced killing despite the lack of coverage predicted by MATS (50). The MATS assay predicts the potential coverage of a strain based on the presence and sero-conservation of the 4 main vaccine antigens expressed in the strain and here we revealed that PorB and LOS responses elicited by the OMV may be responsible for the 4CMenB coverage of these strains. Real world evidence showed that protection conferred by 4CMenB is broader than what is predicted by current typing methods for circulating strains (11, 12) and has the potential to provide some protection beyond MenB disease (19) and cross-protecting responses to OMV antigens such as PorB and LOS may be responsible for some of this.

Multiple post-implementation surveillance studies revealed a decline in gonorrhea rates in subjects immunized with OMV-based meningococcal vaccines, such as 4CMenB and MeNZB, possibly due to cross-protection induced by similar components on the surface of meningococci and gonococci (19, 27, 30–33). Preclinical studies showed that antibodies induced by the OMV-based vaccine recognized gonococcal surface antigens (37) and 4CMenB immunization of mice accelerated clearance of the infection after gonococcal challenge (38). Our results show that despite only moderate homology (67% identity) between PorB3 of the OMV and gonococcus PorB1B, anti-PorB mAbs belonging to Cluster 2, together with the LOS-specific HumAb, could recognize several laboratory and circulating gonococcal strains (both PorB1A and PorB1B) and effect bactericidal activity against FA1090 gonococcus strain. While the serum bactericidal antibody assay is the “gold standard” for measuring serologic protection against *Neisseria meningitidis* (66), there is no such correlate of protection for gonococcus. However, the cross functional activity of these 4CMenB HumAbs suggests that these antigens could be implicated in the cross protection observed after vaccination with 4CMenB or indeed MeNZB against gonococcal infections. Complement-mediated killing has been implicated as important for protection against gonococcus, and the 2C7 mAb to LOS is bactericidal *in vitro* and has been shown to be protective in mouse models (67). The cross functional PorB HumAbs that result bactericidal against gonococcus were all members of a VH4-34 clonal family in cluster 2, all elicited from Sbj1. In a parallel study isolating OMV-specific HumAbs from memory B cells from 4CMenB vaccinees and selecting specifically for gonococcal-specific HumAbs, intriguingly the same 2 antigens PorB and LOS have been identified as the target antigens and all of the functional PorB HumAbs were from the VH4-34 germline (Troisi, Fabbrini et al., 2023, bioRxiv, https://www.biorxiv.org/content/10.1101/2023.12.07.570438v1). Therefore, despite interrogating a distinct B cell set (memory B cells as opposed to plasmablasts) from different subjects and using a distinct screening pipeline (gonococcal-specific mAbs instead of meningococcal-specific) both studies converged on similar results. Interestingly the LOS mAbs isolated from both studies appear to recognize distinct epitopes on the α-chain extended from the Hep1 core, which is within the L3,7,9 LOS immunotype expressed on the meningococcal 4CMenB OMV component. Both LOS and PorB antigens contribute to the ability of gonococci to resist complement-mediated killing through complement negative regulator engagement in gonococcus (68) and therefore targeting immune evasion mechanisms may be an important strategy in cross protection of 4CMenB and future gonococcal interventions.

The LOS HumAb isolated here with cross-functional bactericidal activity binds an epitope consisting of the third and fourth sugars on the α-chain extended from the Hep1 core. Interestingly, this epitope is distinct from the epitope recognized by the bactericidal and protective 2C7 mAb (69), which was isolated from hybridomas after immunization of mice with gonococcus. This epitope has been reported to be commonly present on gonococcus during human infection (8). 2C7 recognizes an epitope engaging the first 2 sugars of both the α-chain and β-chain (58). This suggests that the 4CMenB OMV may elicit cross-functional LOS antibodies and may be distinct from those that are induced by the gonococcus, that can cross react with gonococcal strains expressing similar LOS structures. Furthermore, we show here that the PorB HumAbs responses are multiple and predicted to have been elicited from multiple distinct epitopes, however only the polarized VH4-34 response in individuals leads to cross reactivity with gonococcus. There are some evidence from the literature that anti-LOS and anti-PorB responses in humans may be protective against gonococcus. In a study of human experimental gonococcal infection, male volunteers who mounted an anti-LOS response after gonococcal challenge were relatively resistant to re-infection with the homologous strain supporting a protective role for LOS antibodies (70). In a study in women with recurring gonococcal infections, women subsequently infected with a strain of the same PorB serotype were less likely to develop salpingitis, suggesting that PorB responses may provide serotype-specific protection against ascending gonococcal disease (71). Finally, retrospective analysis on a failed vaccine human challenge trial showed that the ratio of the concentration of PorB and LOS antibodies to that of Rmp antibody (PorB-Ab + LOS-Ab/Rmp-Ab) in the sera of the subjects was positively correlated with protection in both vaccine and placebo recipients (72).

In summary, this study along with others of its type are revealing interesting results on antigens and epitopes elicited by current vaccines such as 4CMenB, towards understanding the full potential for OMV-based meningococcal vaccines to confer broad protection against meningococcal disease and also against *N.gonorrhoeae* infection. Given the lack of correlates of protection against gonorrhea infection and slow progress in vaccine candidates, these findings were both reminiscent of early studies where anti PorB and LOS responses were investigated and intriguing for future gonococcal vaccine design.

## Data Availability

The data presented in this study are deposited in the NCBI’s Gene Expression Omnibus repository, accession numbers GSE291727, GSE291733, GSE291737, GSE291739.

## References

[1] Sierra GV Campa HC Varcacel NM Garcia IL Izquierdo PL Sotolongo PF. Vaccine against group B Neisseria meningitidis: protection trial and mass vaccination results in Cuba. NIPH Ann. (1991) 14:195–207. 1812432

[2] Fredriksen JH Rosenqvist E Wedege E Bryn K Bjune G Frøholm LO. Production, characterization and control of MenB-vaccine “Folkehelsa”: an outer membrane vesicle vaccine against group B meningococcal disease. NIPH Ann. (1991) 14:67–79. 1812438

[3] Boslego J Garcia J Cruz R Zollinger W Brandt B Ruiz S. Efficacy, safety, and immunogenicity of a meningococcal group B (15:P1.3) outer membrane protein vaccine in Iquique, Chile. Chilean National Committee for Meningococcal Disease. Vaccine. (1995) 13:821–9. doi: 10.1016/0264-410X(94)00037-N 7483804

[4] Holst J Feiring B Fuglesang E Høiby EA Nøkleby H Aaberge IS. Serum bactericidal activity correlates with the vaccine efficacy of outer membrane vesicle vaccines against Neisseria meningitidis serogroup B disease. Vaccine. (2003) 21:734–7. doi: 10.1016/S0264-410X(02)00591-1 12531351

[5] Giuliani M Bartolini E Galli B Santini L Lo Surdo P Buricchi F. Human protective response induced by meningococcus B vaccine is mediated by the synergy of multiple bactericidal epitopes. Sci Rep. (2018) 8:3700. doi: 10.1038/s41598-018-22057-7 29487324 PMC5829249

[6] Martin DR Ruijne N McCallum L O’Hallahan J Oster P. The VR2 epitope on the PorA P1.7-2,4 protein is the major target for the immune response elicited by the strain-specific group B meningococcal vaccine MeNZB. Clin Vaccine Immunol. (2006) 13:486–91. doi: 10.1128/CVI.13.4.486-491.2006 PMC1459632 16603616

[7] Findlow J Borrow R Snape MD Dawson T Holland A Tessa MJ. Multicenter, open-label, randomized phase II controlled trial of an investigational recombinant Meningococcal serogroup B vaccine with and without outer membrane vesicles, administered in infancy. Clin Infect Dis. (2010) 51:1127–37. doi: 10.1086/656741 20954968

[8] Snape MD Dawson T Evans A Terssa MJ Ohene-Kena B Findlow J. Immunogenicity of two investigational serogroup B meningococcal vaccines in the first year of life: a randomized comparative trial. Pediatr Infect Dis J. (2010) 29:e71–79. doi: 10.1097/INF.0b013e3181f59f6d 20844462

[9] Esposito S Prymula R Zuccotti GV Xie F Barone M Dull PM. A phase 2 randomized controlled trial of a multicomponent meningococcal serogroup B vaccine, 4CMenB, in infants (II). Hum Vaccin Immunother. (2014) 10:2005–14. doi: 10.4161/hv.29218 PMC4186018 25424810

[10] Donnelly J Medini D Boccadifuoco G Biolchi A Ward J Frasch C. Qualitative and quantitative assessment of meningococcal antigens to evaluate the potential strain coverage of protein-based vaccines. Proc Natl Acad Sci U.S.A. (2010) 107:19490–5. doi: 10.1073/pnas.1013758107 PMC2984153 20962280

[11] Frosi G Biolchi A Lo Sapio M Rigat F Gilchrist S Lucidarme J. Bactericidal antibody against a representative epidemiological meningococcal serogroup B panel confirms that MATS underestimates 4CMenB vaccine strain coverage. Vaccine. (2013) 31:4968–74. doi: 10.1016/j.vaccine.2013.08.006 23954380

[12] Martinón-Torres F Banzhoff A Azzari C De Wals P Marlow R Marshall H. Recent advances in meningococcal B disease prevention: real-world evidence from 4CMenB vaccination. J Infect. (2021) 83:17–26. doi: 10.1016/j.jinf.2021.04.031 33933528

[13] Viviani V Biolchi A Pizza M. Synergistic activity of antibodies in the multicomponent 4CMenB vaccine. Expert Rev Vaccines. (2022) 21:645–58. doi: 10.1080/14760584.2022.2050697 35257644

[14] Matthias KA Reveille A Connolly KL Jerse AE Gao YS Bash MC. Deletion of major porins from meningococcal outer membrane vesicle vaccines enhances reactivity against heterologous serogroup B Neisseria meningitidis strains. Vaccine. (2020) 38:2396–405. doi: 10.1016/j.vaccine.2020.01.038 PMC11656520 32037226

[15] Arnold R Galloway Y McNicholas A O’Hallahan J. Effectiveness of a vaccination programme for an epidemic of meningococcal B in New Zealand. Vaccine. (2011) 29:7100–6. doi: 10.1016/j.vaccine.2011.06.120 21803101

[16] Viviani V Fantoni A Tomei S Marchi S Luzzi E Bodini M. OpcA and PorB are novel bactericidal antigens of the 4CMenB vaccine in mice and humans. NPJ Vaccines. (2023) 8:54. doi: 10.1038/s41541-023-00651-9 37045859 PMC10097807

[17] Biolchi A De Angelis A Moschioni M Tomei S Brunelli B Giuliani M. Multicomponent meningococcal serogroup B vaccination elicits cross-reactive immunity in infants against genetically diverse serogroup C, W and Y invasive disease isolates. Vaccine. (2020) 38:7542–50. doi: 10.1016/j.vaccine.2020.09.050 33036804

[18] Biolchi A Tomei S Brunelli B Giuliani M bambini S Borrow R. 4CMenB immunization induces serum bactericidal antibodies against non-serogroup B meningococcal strains in adolescents. Infect Dis Ther. (2021) 10:307–16. doi: 10.1007/s40121-020-00370-x PMC7954916 33185849

[19] Ruiz García Y Sohn WY Seib KL Taha MK Vázquez JA de Lemos APS. Looking beyond meningococcal B with the 4CMenB vaccine: the Neisseria effect. NPJ Vaccines. (2021) 6:130. doi: 10.1038/s41541-021-00388-3 34716336 PMC8556335

[20] Carter NJ. Multicomponent meningococcal serogroup B vaccine (4CMenB; Bexsero(^®^)): a review of its use in primary and booster vaccination. BioDrugs. (2013) 27:263–74. doi: 10.1007/s40259-013-0029-2 23575646

[21] Hong E Giuliani MM Deghmane AE Comanducci M Brunelli B Dull P. Could the multicomponent meningococcal serogroup B vaccine (4CMenB) control Neisseria meningitidis capsular group X outbreaks in Africa? Vaccine. (2013) 31:1113–6. doi: 10.1016/j.vaccine.2012.12.022 23261039

[22] Rivero-Calle I Raguindin PF Gómez-Rial J Rodriguez-Tenreiro C Martinón-Torres F. Meningococcal group B vaccine for the prevention of invasive meningococcal disease caused by neisseria meningitidis serogroup B. Infect Drug Resist. (2019) 12:3169–88. doi: 10.2147/IDR.S159952 PMC6793463 31632103

[23] Marshall HS Andraweera PH Wang B McMillan M Koehler AP Lally N. Evaluating the effectiveness of the 4CMenB vaccine against invasive meningococcal disease and gonorrhoea in an infant, child and adolescent program: protocol. Hum Vaccin Immunother. (2021) 17:1450–4. doi: 10.1080/21645515.2020.1827614 PMC8078704 33428528

[24] McMillan M Wang B Koehler AP Sullivan TR Marshall HS. Impact of meningococcal B vaccine on invasive meningococcal disease in adolescents. Clin Infect Dis. (2021) 73:e233–7. doi: 10.1093/cid/ciaa1636 33587122

[25] Efron A Biolchi A Pereira CS Tomei S Campos J De Belder D. Bactericidal killing of meningococcal W strains isolated in Argentina by the sera of adolescents and infants immunized with 4-component meningococcal serogroup B vaccine (4CMenB). Hum Vaccin Immunother. (2023) 19:2288389. doi: 10.1080/21645515.2023.2288389 38111094 PMC10732599

[26] Whelan J Kløvstad H Haugen IL Holle MR Storsaeter J. Ecologic study of meningococcal B vaccine and neisseria gonorrhoeae infection, Norway. Emerg Infect Dis. (2016) 22:1137–9. doi: 10.3201/eid2206.151093 PMC4880101 27191543

[27] Petousis-Harris H Paynter J Morgan J Saxton P McArdle B Goodyear-Smith F. Effectiveness of a group B outer membrane vesicle meningococcal vaccine against gonorrhoea in New Zealand: a retrospective case-control study. Lancet. (2017) 390:1603–10. doi: 10.1016/S0140-6736(17)31449-6 28705462

[28] Reyes Díaz LM Lastre González MSJB Cuello M Sierra-González VG Pupo RR Lantero MI. VA-MENGOC-BC vaccination induces serum and mucosal anti neisseria gonorrhoeae immune responses and reduces the incidence of gonorrhea. Pediatr Infect Dis J. (2021) 40:375–81. doi: 10.1097/INF.0000000000003047 33591079

[29] Raccagni AR Diotallevi S Lolatto R Bruzzesi E Martearena Garcia MC Mainardi I. Breakthrough rectal neisseria gonorrhoeae infections after meningococcal B vaccination: microbiological and clinical features. Open Forum Infect Dis. (2024) 11:ofae562. doi: 10.1093/ofid/ofae562 39498171 PMC11532644

[30] Wang B Giles L Andraweera P McMillan M Almond S Beazley R. Effectiveness and impact of the 4CMenB vaccine against invasive serogroup B meningococcal disease and gonorrhoea in an infant, child, and adolescent programme: an observational cohort and case-control study. Lancet Infect Dis. (2022) 22:1011–20. doi: 10.1016/S1473-3099(21)00754-4 35427492

[31] Abara WE Bernstein KT Lewis FMT Schillinger JA Feemster K Pathela P. Effectiveness of a serogroup B outer membrane vesicle meningococcal vaccine against gonorrhoea: a retrospective observational study. Lancet Infect Dis. (2022) 22:1021–9. doi: 10.1016/S1473-3099(21)00812-4 PMC10227473 35427490

[32] Bruxvoort KJ lewnard JA Cheng LH Tseng HF Chang J Veltman J. Prevention of neisseria gonorrhoeae with meningococcal B vaccine: A matched cohort study in southern california. Clin Infect Dis. (2023) 76:e1341–9. doi: 10.1093/cid/ciac436 35642527

[33] Raccagni AR Galli L Spagnuolo V Bruzzesi E Muccini C Bossolasco S. Meningococcus B vaccination effectiveness against neisseria gonorrhoeae infection in people living with HIV: A case-control study. Sex Transm Dis. (2023) 50:247–51. doi: 10.1097/OLQ.0000000000001771 36728240

[34] Marjuki H Topaz N Joseph SJ Gernert KM Kersh EN. Genetic similarity of gonococcal homologs to meningococcal outer membrane proteins of serogroup B vaccine. mBio. (2019) 10. doi: 10.1128/mBio.01668-19 PMC6737241 31506309

[35] Semchenko EA Tan A Borrow R Seib KL. The serogroup B meningococcal vaccine bexsero elicits antibodies to neisseria gonorrhoeae. Clin Infect Dis. (2019) 69:1101–11. doi: 10.1093/cid/ciy1061 PMC6743822 30551148

[36] Leduc I Connolly KL Begum A Underwood K Darnnell S Shafer WM. The serogroup B meningococcal outer membrane vesicle-based vaccine 4CMenB induces cross-species protection against Neisseria gonorrhoeae. PLoS Pathog. (2020) 16:e1008602. doi: 10.1371/journal.ppat.1008602 33290434 PMC7748408

[37] Stejskal L Thistlethwaite A Ramirez-Bencomo F Rashmi S Harrison O Feavers IM. Profiling IgG and IgA antibody responses during vaccination and infection in a high-risk gonorrhoea population. Nat Commun. (2024) 15:6712. doi: 10.1038/s41467-024-51053-x 39112489 PMC11306574

[38] Tzeng YL Sannigrahi S Borrow R Stephens DS. Neisseria gonorrhoeae lipooligosaccharide glycan epitopes recognized by bactericidal IgG antibodies elicited by the meningococcal group B-directed vaccine, MenB-4C. Front Immunol. (2024) 15:1350344. doi: 10.3389/fimmu.2024.1350344 38440731 PMC10909805

[39] Rappuoli R Bottomley MJ D’Oro U Finco O De Gregorio E. Reverse vaccinology 2.0: Human immunology instructs vaccine antigen design. J Exp Med. (2016) 213:469–81. doi: 10.1084/jem.20151960 PMC4821650 27022144

[40] Bidmos FA Siris S Gladstone CA Langford PR. Bacterial vaccine antigen discovery in the reverse vaccinology 2.0 era: progress and challenges. Front Immunol. (2018) 9:2315. doi: 10.3389/fimmu.2018.02315 30349542 PMC6187972

[41] De Jong RN Beurskens FJ Verploegen S Strumane K Van Kampen MD Voorhorst M. A novel platform for the potentiation of therapeutic antibodies based on antigen-dependent formation of igG hexamers at the cell surface. PLoS Biol. (2016) 14:e1002344. doi: 10.1371/journal.pbio.1002344 26736041 PMC4703389

[42] Clargo AM Hudson AR Ndlovu W Wootton RJ Cremin LA O’Dowd VL. The rapid generation of recombinant functional monoclonal antibodies from individual, antigen-specific bone marrow-derived plasma cells isolated using a novel fluorescence-based method. MAbs. (2014) 6:143–59. doi: 10.4161/mabs.27044 PMC3929438 24423622

[43] Giuliani MM Adu-Bobie J Comanducci M Aricò B Savino S Santini L. A universal vaccine for serogroup B meningococcus. Proc Natl Acad Sci U S A. (2006) 103:10834–9. doi: 10.1073/pnas.0603940103 PMC2047628 16825336

[44] Ye J Ma N Madden TL Ostell JM. IgBLAST: an immunoglobulin variable domain sequence analysis tool. Nucleic Acids Res. (2013) 41:W34–40. doi: 10.1093/nar/gkt382 PMC3692102 23671333

[45] Lefranc M-P Pommié C Ruiz M Giudicelli V Foulquier E Truong L. IMGT unique numbering for immunoglobulin and T cell receptor variable domains and Ig superfamily V-like domains. Dev Comp Immunol. (2003) 27:55–77. doi: 10.1016/S0145-305X(02)00039-3 12477501

[46] Galson JD Clutterbuck EA Trück J Ramasamy MN Münz M Fowler A. BCR repertoire sequencing: different patterns of B-cell activation after two Meningococcal vaccines. Immunol Cell Biol. (2015) 93:885–95. doi: 10.1038/icb.2015.57 PMC4551417 25976772

[47] Ruffolo JA Sulam J Gray JJ. Antibody structure prediction using interpretable deep learning. Patterns (N Y). (2022) 3:100406. doi: 10.1016/j.patter.2021.100406 35199061 PMC8848015

[48] Dominguez C Boelens R Bonvin AM. HADDOCK: a protein-protein docking approach based on biochemical or biophysical information. J Am Chem Soc. (2003) 125:1731–7. doi: 10.1021/ja026939x 12580598

[49] Chinery L Wahome N Moal I Deane CM. Paragraph—antibody paratope prediction using graph neural networks with minimal feature vectors. Bioinformatics. (2022) 39. doi: 10.1021/ja026939x 36370083

[50] Efron A Brozzi A Biolchi A Bodini M Giuliani M Guidotti S. Genetic characterization and estimated 4CMenB vaccine strain coverage of 284 Neisseria meningitidis isolates causing invasive meningococcal disease in Argentina in 2010-2014. Hum Vaccin Immunother. (2024) 20:2378537. doi: 10.1080/21645515.2024.2378537 39037011 PMC11789736

[51] Massari P King CA MacLeod H Wetzler LM. Improved purification of native meningococcal porin PorB and studies on its structure/function. Protein Expression Purification. (2005) 44:136–46. doi: 10.1016/j.pep.2005.04.021 16027004

[52] Tanabe M Nimigean CM Iverson TM. Structural basis for solute transport, nucleotide regulation, and immunological recognition of *Neisseria meningitidis* PorB. Proc Natl Acad Sci. (2010) 107:6811–6. doi: 10.1073/pnas.0912115107 PMC2872391 20351243

[53] Bartsch A Ives CM Kattner C Pein F Diehn M Tanabue M. An antibiotic-resistance conferring mutation in a neisserial porin: Structure, ion flux, and ampicillin binding. Biochim Biophys Acta Biomembr. (2021) 1863:183601. doi: 10.1016/j.bbamem.2021.183601 33675718 PMC8047873

[54] Chinery L Wahome N Moal I Deane CM. Paragraph-antibody paratope prediction using graph neural networks with minimal feature vectors. Bioinformatics. (2023) 39. doi: 10.1093/bioinformatics/btac732 36370083

[55] Mandrell RE Griffiss JM Macher BA. Lipooligosaccharides (LOS) of Neisseria gonorrhoeae and Neisseria meningitidis have components that are immunochemically similar to precursors of human blood group antigens. Carbohydrate sequence specificity of the mouse monoclonal antibodies that recognize crossreacting antigens on LOS and human erythrocytes. J Exp Med. (1988) 168:107–26. doi: 10.1084/jem.168.1.107 PMC2188965 2456365

[56] Mubaiwa TD Hartley-Tassell LE Semchenko EA Jen ECF Srikhanta TN Day CJ. The glycointeractome of serogroup B Neisseria meningitidis strain MC58. Sci Rep. (2017) 7:5693. doi: 10.1038/s41598-017-05894-w 28720847 PMC5515891

[57] Jennings MP Srikhanta YN Moxon ER Kramer M Poolman JT Kuipers B. The genetic basis of the phase variation repertoire of lipopolysaccharide immunotypes in Neisseria meningitidis. Microbiol (Reading). (1999) 145:3013–21. doi: 10.1099/00221287-145-11-3013 10589709

[58] Chakraborti S Lewis LA Cox AD St Michael F Li J Rice PA. Phase-Variable Heptose I Glycan Extensions Modulate Efficacy of 2C7 Vaccine Antibody Directed against Neisseria gonorrhoeae Lipooligosaccharide. J Immunol. (2016) 196:4576–86. doi: 10.4049/jimmunol.1600374 PMC4875794 27183633

[59] Waltmann A Chen JS Duncan JA. Promising developments in gonococcal vaccines. Curr Opin Infect Dis. (2024) 37:63–9. doi: 10.1097/QCO.0000000000000992 PMC11625492 38050729

[60] Awanye AM Chang CM Wheeler JX Chan H Marsay L Dold C. Immunogenicity profiling of protein antigens from capsular group B Neisseria meningitidis. Sci Rep. (2019) 9:6843. doi: 10.1038/s41598-019-43139-0 31048732 PMC6497663

[61] Findlow J Lucidarme J Taha MK Burman C Balmer P. Correlates of protection for meningococcal surface protein vaccines: lessons from the past. Expert Rev Vaccines. (2022) 21:739–51. doi: 10.1080/14760584.2021.1940144 34287103

[62] Tani C Stella M Donnarumma D Biagini M Parente P Vada A. Quantification by LC-MS(E) of outer membrane vesicle proteins of the Bexsero^®^ vaccine. Vaccine. (2014) 32:1273–9. doi: 10.1016/j.vaccine.2014.01.011 24462403

[63] Natali EN Principato S Ferlicca F Bianchi F Fontana LE Faleri A. Synergic complement-mediated bactericidal activity of monoclonal antibodies with distinct specificity. FASEB J. (2020) 34:10329–41. doi: 10.1096/fj.201902795R 32725956

[64] Michaelsen TE Aase A Kolberg J Wedge E Rosenqvist E. PorB3 outer membrane protein on Neisseria meningitidis is poorly accessible for antibody binding on live bacteria. Vaccine. (2001) 19:1526–33. doi: 10.1016/S0264-410X(00)00324-8 11163678

[65] Kandler JL Holley CL Reimche JL Dhulipala V Balthazar JT Muszyński A. The misR response regulator is necessary for intrinsic cationic antimicrobial peptide and aminoglycoside resistance in neisseria gonorrhoeae. Antimicrob Agents Chemother. (2016) 60:4690–700. doi: 10.1128/AAC.00823-16 PMC4958169 27216061

[66] Goldschneider I Gotschlich EC Artenstein MS. Human immunity to the meningococcus. II. Development of natural immunity. J Exp Med. (1969) 129:1327–48. doi: 10.1084/jem.129.6.1327 PMC2138665 4977281

[67] Gulati S Beurskens FJ De Kreuk B-J Roza M Zheng B DeOliveira RB. Complement alone drives efficacy of a chimeric antigonococcal monoclonal antibody. PLoS Biol. (2019) 17:e3000323. doi: 10.1371/journal.pbio.3000323 31216278 PMC6602280

[68] Lewis LA and Ram S. Complement interactions with the pathogenic Neisseriae: clinical features, deficiency states, and evasion mechanisms. FEBS Lett. (2020) 594:2670–94. doi: 10.1002/1873-3468.13760 32058583

[69] Gulati S McQuillen DP Mandrell RE Jani DB Rice PA. Immunogenicity of Neisseria gonorrhoeae lipooligosaccharide epitope 2C7, widely expressed *in vivo* with no immunochemical similarity to human glycosphingolipids. J Infect Dis. (1996) 174:1223–37. doi: 10.1093/infdis/174.6.1223 8940213

[70] Schmidt KA Schneider H Lindstrom JA Boslego JW Warren R Van De Verg L. Experimental gonococcal urethritis and reinfection with homologous gonococci in male volunteers. Sex Transm Dis. (2001) 28:555–64. doi: 10.1097/00007435-200110000-00001 11689753

[71] Buchanan TM Eschenbach DA Knapp JS Holmes KK. Gonococcal salpingitis is less likely to recur with Neisseria gonorrhoeae of the same principal outer membrane protein antigenic type. Am J Obstet Gynecol. (1980) 138:978–80. doi: 10.1016/0002-9378(80)91091-1 6781350

[72] Rice PA Shafer WM Ram S Jerse AE. Neisseria gonorrhoeae: drug resistance, mouse models, and vaccine development. Annu Rev Microbiol. (2017) 71:665–86. doi: 10.1146/annurev-micro-090816-093530 28886683

