# Supplementary material for: Isolation of human monoclonal antibodies from 4CMenB vaccinees reveals PorB and LOS as the main OMV components inducing cross-strain protection
Source: Front Immunol. 2025 Apr 16;16:1565862. doi: 10.3389/fimmu.2025.1565862 (PMC12040683; doi:10.3389/fimmu.2025.1565862)
Supplement: Supplementary file 1 [file DataSheet1.pdf]

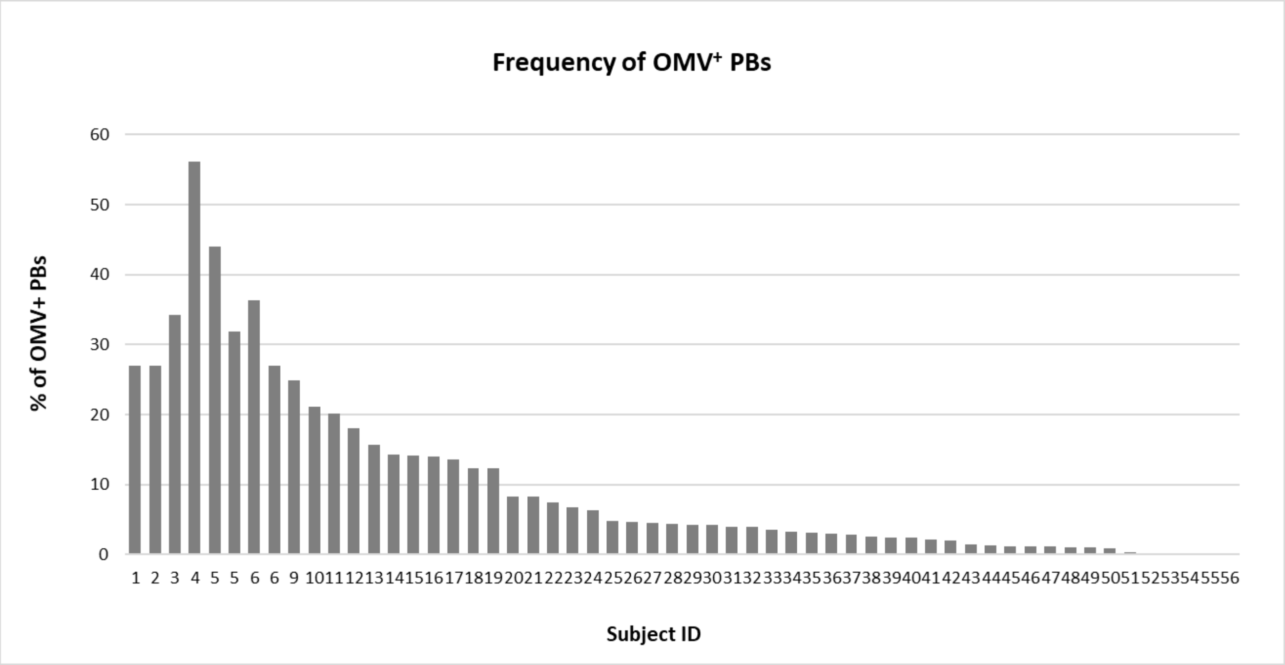


**Supplementary Figure 1. Reactivity of PBMCs from vaccinees to OMV stimulation analysed in an ELISpot assay.** Histogram reporting for each subject the frequency of IgG-secreting Plasmablasts in response to OMV stimulation, represented as % of OMV-responsive PBs on the total IgG-secreting population of PBs.

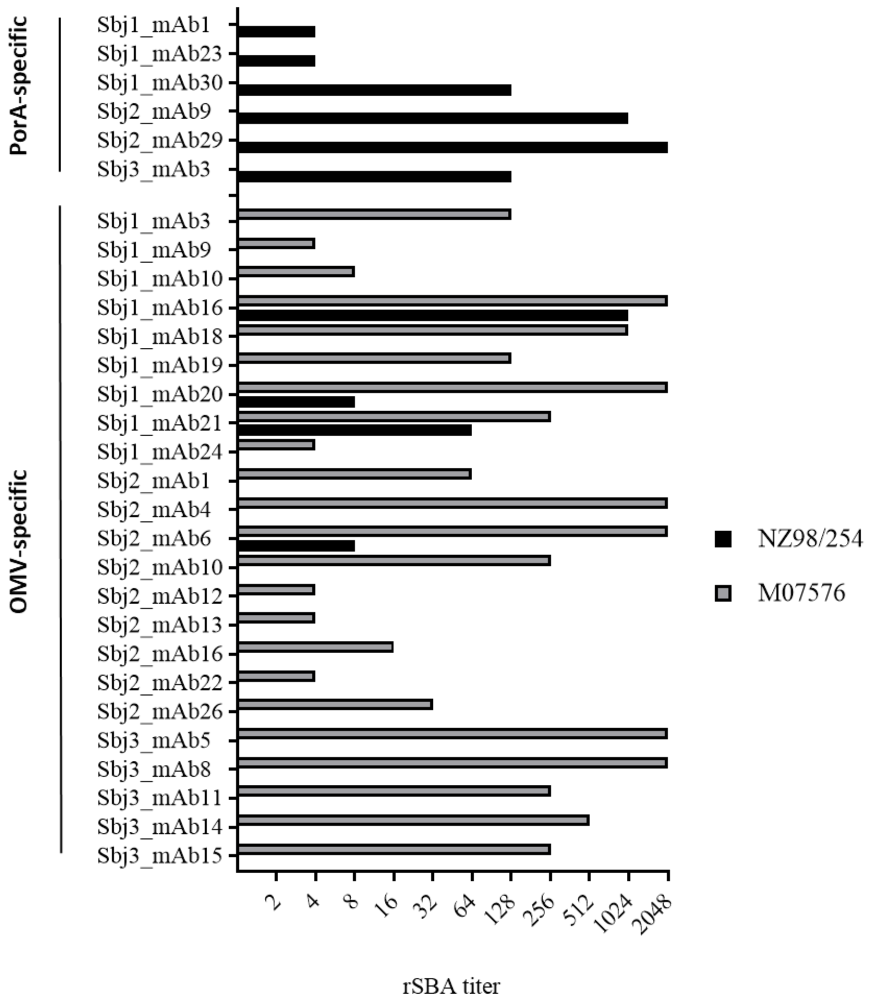


**Supplementary Figure 2. Bactericidal activity on 2 MenB strains of TAP-expressed HumAbs in raw supernatant.** rSBA titers of bactericidal OMV- and PorA-specific HumAbs expressed in small scale and tested as raw supernatants. On the X axis is reported the reciprocal of the highest supernatant dilution at which HumAbs showed bactericidal activity. On the Y axis is reported the name of each tested mAb on the NZ98/254 (black bars) and M07576 (grey bars) MenB strains. HumAbs are represented grouped per specificity, with the HumAbs defined as PorA-specific by the Luminex binding assay represented on the upper part and the HumAbs defined as OMV-specific HumAbs represented on the lower part of the graph.

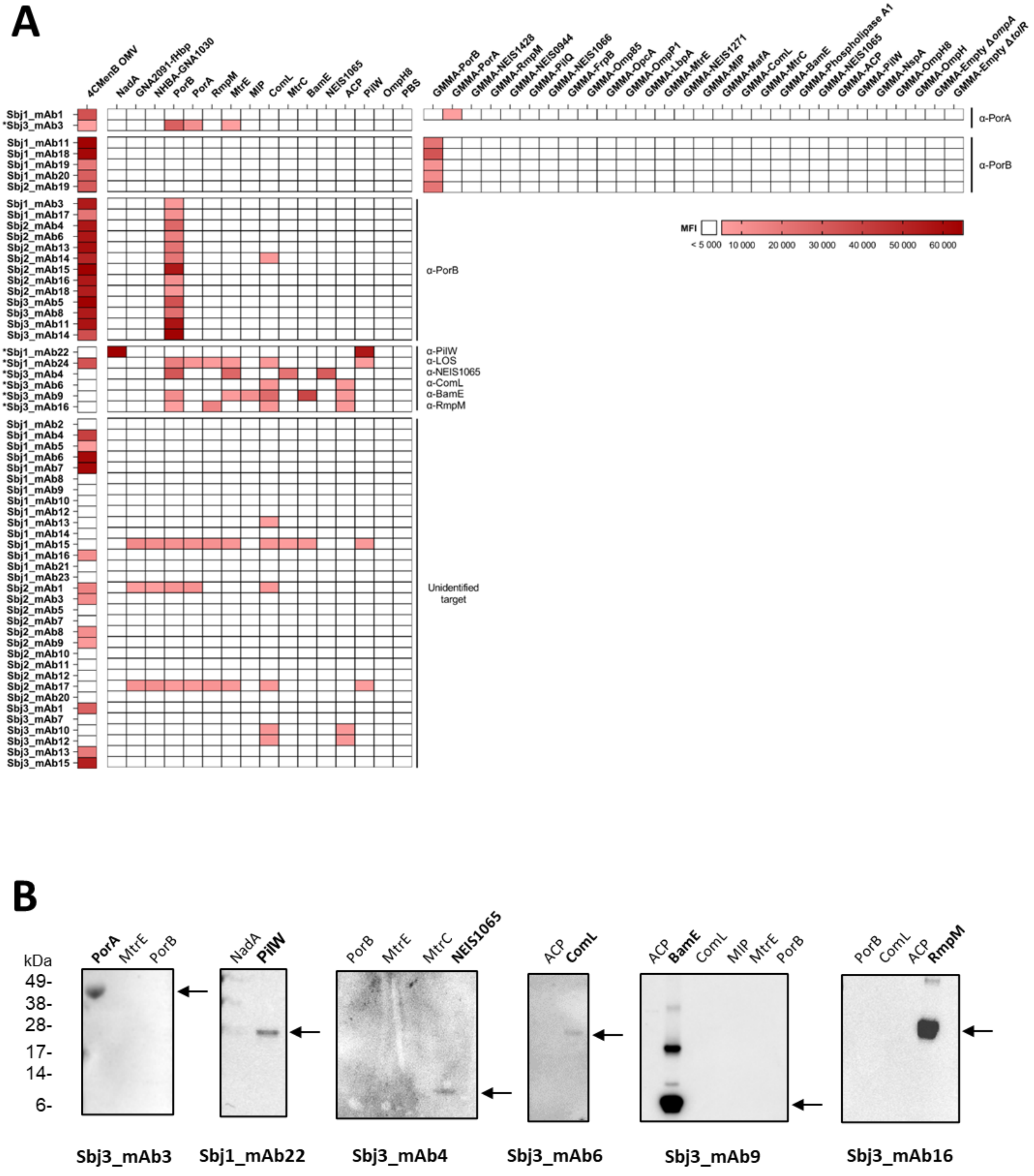


**Supplementary Figure 3. Binding characterization of HumAbs. A)** Heatmap representing the reactivity (Mean Fluorescence Intensity, MFI) of the 58 purified HumAbs against a selected panel of MenB proteins expressed recombinantly in *E.coli* (left panel) and/or on *E.coli* GMMA (right panel). White boxes represent values below cut-off of positivity (MFI < 5000), asterisk (*) identify HumAbs with multiple targets that have been further characterized in Western blot. **B)** Western blots of selected HumAbs on the recombinant proteins recognized on the protein array.

| Reaction step | | Primer ID | Primer sequence |
|---|---|---|---|
| RT-PCR | | IgG | GGAAGGTGTGCACGCCGCTGGTC |
| | | IgA | CCTGGGGGAAGAAGCCCTGGACC |
| | | IgM | GGGAATTCTCACAGGAGACGA |
| | | Cκ | CCTCTAACACTCTCCCCTGTTGAAG |
| | | Cλ | CATTCTGYAGGGGCMACTGTCTTCTC |
| PCR1 | VH | Fw_VH1_VH7 | CACTCCCAGGTGCAGCTGGTGCAG |
| | | Fw_VH2 | TGGGTCTTRTCCCAGGTCACCTTG |
| | | Fw_VH 3 | AAGGTGTCCAGTGTSAGGTGCAG |
| | | Fw_VH4_6 | GTCCTGTCCCAGGTGCAGCTGCAG |
| | | Fw_VH5 | GAGTCTGTTCCGAGGTGCAGCTGG |
| | | Rv_IgG CH | GTGCCAGGGGGAAGACCGATG |
| | | Rv_IgA CH | GCMGAGGCTCAGCGGGAAGAC |
| | | Rv_IgM CH | GAGACGAGGGGGAAAAGGGTTG |
| | Vκ | Fw_VK1 | CAGGTGCCAGATGTGHCATCCAG |
| | | Fw_VK2 | CTGGATCCAGTGSGGATATTGTGATG |
| | | Fw_VK3 | CCCAGATACCACCGGAGAAATTGTG |
| | | Fw_VK4 | CTCTGGTGCCTACGGGGACATCGTG |
| | | Fw_VK5 | CTGATACCAGGGCAGAAACGACAC |
| | | Rv_CK | GAACACTCTCCCCTGTTGAAGCTCTTTG |
| | Vλ | Fw_VL1 | GGTCCTGGGCCCAGTCTGTGCTG |
| | | Fw_VL2 | GGTCCTGGGCCCAGTCTGCCCTG |
| | | Fw_VL3 | TCTGTGRCCTCCTATGAGCTGAC |
| | | Fw_VL4_VL5_VL9 | CTCTCGCAGCCTGTGCTGACTCA |
| | | Fw_VL6 | GTTCTTGGGCCAATTTTATGCTG |
| | | Fw_VL7 | GGTCCAATTCTCAGGCTGTGGTG |
| | | Fw_VL8 | GAGTGGATTCTCAGACTGTGGTG |
| | | Fw_VL10 | GTCAGTGGTCCAGGCAGGGCTGAC |
| | | Rv_CL | GTGCTCCCTTCATGCGTGACC |
| PCR2 | VH | Fw_VH1_5_7 | CCTGGTCGCGATCCTGGAAGGCGTGCACTGCCAGGTGCAGCTGGTGCAGTCTG |
| | | Fw_VH2 | CCTGGTCGCGATCCTGGAAGGCGTGCACTGCCAGGTCACCTTGAAGGAGTCTGGTC |
| | | Fw_VH3 | CCTGGTCGCGATCCTGGAAGGCGTGCACTGCGAGGTGCAGCTGGTGGAGTCTGGGGGAG |
| | | Fw_VH4_6_a | CCTGGTCGCGATCCTGGAAGGCGTGCACTGCCAGGTGCAGCTGCAGGAGTCGGG |
| | | Fw_VH4_6_b | CCTGGTCGCGATCCTGGAAGGCGTGCACTGCCAGGTGCAGCTGCAGCAGTGGGG |
| | | Rv_IgG CH | CTGCTGGGGGCCAGGGGGAACACGCTTGGGCCCTTGGTGGARGC |
| | | Rv_IgA CH | CTGCTGGGGGCCAGGGGGAACACGCTCTTGGGGCTGGTCGGGGA |
| | | Rv_IgM CH | CTGCTGGGGGCCAGGGGGAACACGCTTGGGGCGGATGCACTCCC |
| | | Fw_PIPE | AGCGTGTTCCCCCTGGCCCCCAGCAGCAAGAGCACCAGCGGCGGCACAGCC |
| | | Rv_PIPE | GCAGTGCACGCCTTCCAGGATCGCGACCAGGAACACCCAGCTCAGGCCGA |
| | Vk | Fw_VK1 | GCTGCTGCTGTGGCTTCCGGACACCACCGGTGCCATCCAGATGACCCAGTCTCCATC |
| | | Fw_VK2_a | GCTGCTGCTGTGGCTTCCGGACACCACCGGTGATATTGTGATGACCCAGACTCCACTCTC |
| | | Fw_VK2_b | GCTGCTGCTGTGGCTTCCGGACACCACCGGTGATATTGTGATGACTCAGTCTCCACTCTC |
| | | Fw_VK3_a | GCTGCTGCTGTGGCTTCCGGACACCACCGGTGAAATTGTGTTGACACAGTCTCCAG |
| | | Fw_VK3_b | GCTGCTGCTGTGGCTTCCGGACACCACCGGTGAAATTGTGATGACGCAGTCTCCAG |
| | | Fw_VK4 | GCTGCTGCTGTGGCTTCCGGACACCACCGGTGACATCGTGATGACCCAGTCTCCAG |
| | | Fw_VK5 | GCTGCTGCTGTGGCTTCCGGACACCACCGGTGAAACGACACTCACGCAGTCTCCAG |
| | | Rv_VK | GGCCTGCAGTTCCTCGGAGCTGGGGGGGAAGATGAAGACAGATGGTGCAGCCACAGTTC |
| | | Fw_PIPE | TTCCCCCCCAGCGACGAGCAGCTGAAGAGCGGCACCGCCAGCGTGGTGTG |
| | | Rv_PIPE | ACCGGTGGTGTCCGGAAGCCACAGCAGCAGCAGGAACAGCAGCTCGGCAG |
| | Vλ | Fw_VL1 | GCTGCTGCTGTGGCTTCCGGACACCACCGGTCAGTCTGTGCTGACTCAGCCGCCCTCAG |
| | | Fw_VL2 | GCTGCTGCTGTGGCTTCCGGACACCACCGGTCAGTCTGCCCTGACTCAGCCTGCCTCCG |
| | | Fw_VL3_a | GCTGCTGCTGTGGCTTCCGGACACCACCGGTTCCTATGAGCTGACACAGCCAC |
| | | Fw_VL3_b | GCTGCTGCTGTGGCTTCCGGACACCACCGGTTCCTATGAGCTGACTCAGGACC |
| | | Fw_VL4 | GCTGCTGCTGTGGCTTCCGGACACCACCGGTCAGCCTGTGCTGACTCAATCGTCCTCTG |
| | | Fw_VL5-9 | GCTGCTGCTGTGGCTTCCGGACACCACCGGTCAGCCTGTGCTGACTCAGCCRACTTC |
| | | Fw_VL6 | GCTGCTGCTGTGGCTTCCGGACACCACCGGTAATTTTATGCTGACTCAGCCCCACTC |
| | | Fw_VL7 | GCTGCTGCTGTGGCTTCCGGACACCACCGGTCAGGCTGTGGTGACTCAGGAGCCCTC |
| | | Fw_VL8 | GCTGCTGCTGTGGCTTCCGGACACCACCGGTCAGACTGTGGTGACCCAGGAGCCATC |
| | | Fw_VL10 | GCTGCTGCTGTGGCTTCCGGACACCACCGGTCAGGCAGGGCTGACTCAGCCACCCTCGG |
| | | Rv_CL1 | GGCCTGCAGTTCCTCGGAGCTGGGGGGGAACAGAGTGACCGTGGGGTTGGCCTTGGGCTGACC |
| | | Rv_CL2 | GGCCTGCAGTTCCTCGGAGCTGGGGGGGAACAGAGTGACCGAGGGGGCAGCCTTGGGCTGACC |
| | | Fw_PIPE | TTCCCCCCCAGCTCCGAGGAACTGCAGGCCAACAAGGCCACCCTGGTGTG |
| | | Rv_PIPE | ACCGGTGGTGTCCGGAAGCCACAGCAGCAGCAGGAACAGCAGCTCGGCAG |
| TAP | | Fw_TAP | ATGTACATTTATATTGGCTCATGTC |
| | | Rv_TAP | CCGCCTCAGAAGCCATAGAG |

**Supplementary Table 1. List of primers used in this work.** Sequences of primers used to amplify the variable regions of the heavy and light chain of mAbs encoded in sorted PBs are reported. Primers indicated as RT-PCR are primers used for retrotranscription. Primers indicated as PCR1 are primers used for the first amplification step, primers indicated as PCR2 are primers used for the nested PCR. Regions of complementarity with the plasmid scaffold are underlined.

| mAb ID | MFI on NZ98/254 OMV | Range of mAb concentration [ng/ul] | Selected for further characterization |
|---|---|---|---|
| Sbj1_mAb1 | 32214 | 10 to 20 | Y |
| Sbj1_mAb2 | 32637 | 10 to 20 | Y |
| Sbj1_mAb3 | 32638 | 10 to 20 | Y |
| Sbj1_mAb4 | 27695 | 10 to 20 | Y |
| Sbj1_mAb5 | 639 | <1 | Y |
| Sbj1_mAb6 | 874 | 5 to 10 | Y |
| Sbj1_mAb7 | 33149 | >20 | Y |
| Sbj1_mAb8 | 44356 | >20 | Y |
| Sbj1_mAb9 | 11647 | >20 | Y |
| Sbj1_mAb10 | 153731 | >20 | Y |
| Sbj1_mAb11 | 31055 | <1 | Y |
| Sbj1_mAb12 | 1234 | 5 to 10 | Y |
| Sbj1_mAb13 | 1341 | 5 to 10 | Y |
| Sbj1_mAb14 | 1345 | 5 to 10 | Y |
| Sbj1_mAb15 | 1114 | 5 to 10 | Y |
| Sbj1_mAb16 | 122587 | 10 to 20 | Y |
| Sbj1_mAb17 | 36935 | 5 to 10 | Y |
| Sbj1_mAb18 | 121158 | >20 | Y |
| Sbj1_mAb19 | 107748 | 5 to 10 | Y |
| Sbj1_mAb20 | 107741 | 10 to 20 | Y |
| Sbj1_mAb21 | 107936 | 10 to 20 | Y |
| Sbj1_mAb22 | 36849 | >20 | Y |
| Sbj1_mAb23 | 8363 | 10 to 20 | Y |
| Sbj1_mAb24 | 102427 | 10 to 20 | Y |
| Sbj1_mAb25 | 15894 | 5 to 10 | Y |
| Sbj1_mAb26 | 1479 | >20 | N |
| Sbj1_mAb27 | 1627 | >20 | N |
| Sbj1_mAb28 | 704 | 10 to 20 | N |
| Sbj1_mAb29 | 1219 | 10 to 20 | N |
| Sbj1_mAb30 | 628 | 10 to 20 | N |
| Sbj1_mAb31 | 94934 | 10 to 20 | Y |
| Sbj1_mAb32 | 2214 | >20 | N |
| Sbj1_mAb33 | 820 | >20 | N |
| Sbj1_mAb34 | 1669 | 10 to 20 | N |
| Sbj1_mAb35 | 530 | 10 to 20 | N |
| Sbj1_mAb36 | 690 | 10 to 20 | N |
| Sbj1_mAb37 | 7530 | 5 to 10 | Y |
| Sbj1_mAb38 | 31929 | >20 | Y |
| Sbj2_mAb1 | 3280 | 10 to 20 | N |
| Sbj2_mAb2 | 76440 | 10 to 20 | Y |
| Sbj2_mAb3 | 3265 | 10 to 20 | N |
| Sbj2_mAb4 | 103888 | 5 to 10 | Y |
| Sbj2_mAb5 | 1164 | 10 to 20 | N |
| Sbj2_mAb6 | 110142 | 10 to 20 | Y |
| Sbj2_mAb7 | 3654 | 10 to 20 | N |
| Sbj2_mAb8 | 6211 | 1 to 5 | Y |
| Sbj2_mAb9 | 77009 | 10 to 20 | Y |
| Sbj2_mAb10 | 76176 | 10 to 20 | Y |
| Sbj2_mAb11 | 6623 | 10 to 20 | Y |
| Sbj2_mAb12 | 13488 | 5 to 10 | Y |
| Sbj2_mAb13 | 6185 | 10 to 20 | Y |
| Sbj2_mAb14 | 12534 | 10 to 20 | Y |
| Sbj2_mAb15 | 33104 | 10 to 20 | Y |
| Sbj2_mAb16 | 33349 | 5 to 10 | Y |
| Sbj2_mAb17 | 1953 | 5 to 10 | Y |
| Sbj2_mAb18 | 67483 | 5 to 10 | Y |
| Sbj2_mAb19 | 11653 | >20 | Y |
| Sbj2_mAb20 | 4209 | 5 to 10 | N |
| Sbj2_mAb21 | 2321 | 10 to 20 | N |
| Sbj2_mAb22 | 4146 | 10 to 20 | Y |
| Sbj2_mAb23 | 1833 | >20 | N |
| Sbj2_mAb24 | 580 | >20 | N |
| Sbj2_mAb25 | 1268 | >20 | N |
| Sbj2_mAb26 | 4576 | 5 to 10 | Y |
| Sbj2_mAb27 | 515 | 10 to 20 | N |
| Sbj2_mAb28 | 613 | 10 to 20 | N |
| Sbj2_mAb29 | 89804 | 10 to 20 | Y |
| Sbj2_mAb30 | 581 | 10 to 20 | N |
| Sbj2_mAb31 | 1466 | 10 to 20 | N |
| Sbj2_mAb32 | 1104 | 10 to 20 | N |
| Sbj2_mAb33 | 643 | 10 to 20 | N |
| Sbj2_mAb34 | 500 | 10 to 20 | N |
| Sbj2_mAb35 | 18272 | 5 to 10 | Y |
| Sbj2_mAb36 | 4386 | >20 | N |
| Sbj2_mAb37 | 3972 | 5 to 10 | Y |
| Sbj2_mAb38 | 1383 | >20 | N |
| Sbj2_mAb39 | 1299 | >20 | N |
| Sbj2_mAb40 | 3616 | >20 | N |
| Sbj2_mAb41 | 598 | 10 to 20 | N |
| Sbj3_mAb1 | 895 | 5 to 10 | Y |
| Sbj3_mAb2 | 9552 | 5 to 10 | Y |
| Sbj3_mAb3 | 29739 | 10 to 20 | Y |
| Sbj3_mAb4 | 12863 | 1 to 5 | Y |
| Sbj3_mAb5 | 27261 | 10 to 20 | Y |
| Sbj3_mAb6 | 7183 | 10 to 20 | Y |
| Sbj3_mAb7 | 28677 | 10 to 20 | Y |
| Sbj3_mAb8 | 28567 | >20 | Y |
| Sbj3_mAb9 | 1906 | 10 to 20 | Y |
| Sbj3_mAb10 | 852 | 10 to 20 | N |
| Sbj3_mAb11 | 31292 | >20 | Y |
| Sbj3_mAb12 | 1273 | 1 to 5 | Y |
| Sbj3_mAb13 | 35804 | 10 to 20 | Y |
| Sbj3_mAb14 | 42030 | 10 to 20 | Y |
| Sbj3_mAb15 | 31294 | >20 | Y |
| Sbj3_mAb16 | 2167 | 10 to 20 | Y |
| Sbj3_mAb17 | 27921 | 10 to 20 | Y |
| Sbj3_mAb18 | 880 | 10 to 20 | N |
| Sbj3_mAb19 | 1766 | >20 | N |
| Sbj3_mAb20 | 28864 | >20 | Y |
| Sbj3_mAb21 | 23171 | >20 | Y |

**Supplementary Table 2. Luminex analysis of HumAbs concentration in the raw supernatant and binding on OMV.** For each HumAb is reported the MFI value on NZ98/254 OMV, the estimate concentration in the raw supernatant and the outcome of selection for further characterizations (Y=selected; N= non selected)

| Strain alias | Serogroup | Clonal complex | ST | Year of collection | Country of collection | PorA VR1 | PorA VR2 | fHbp | NHBA | NadA | Reference |
|---|---|---|---|---|---|---|---|---|---|---|---|
| NZ98/254 | B | ST-41/44 complex/Lineage 3 | 42 | 1998 | NZL | 7-2 | 4 | 1.14 | 2 | no | Muzzi A. et al., mSphere 2022; Viviani V. et al., NPJ Vaccines 2023 |
| M07 0241084 | B | ST-41/44 complex/Lineage 3 | 1097 | 2007 | GBR | 19 | 15 | 2,553 | 31 | no | Viviani V. et al., NPJ Vaccines 2023 |
| M13520 | B | ST-41/44 complex/Lineage 3 | 43 | 2005 | USA | 19 | 15-1 | 2.19 | 2 | no | Muzzi A. et al., mSphere 2022 |
| M07576 | B | ST-35 complex | 35 | 2000 | USA | 22-1 | 14 | 2.16 | 21 | no | Muzzi A. et al., mSphere 2022; Viviani V. et al., NPJ Vaccines 2023 |
| M07463 | B | ST-41/44 complex/Lineage 3 | 2851 | 2000 | USA | 17 | 16-3 | 2.24 | 10 | no | Muzzi A. et al., mSphere 2023 |
| M13547 | B | ST-41/44 complex/Lineage 3 | 136 | 2005 | USA | 17 | 16-3 | 2.24 | 11 | no | Muzzi A. et al., mSphere 2023 |
| M09929 | B | ST-35 complex | 3592 | 2002 | USA | 12-1 | 16 | 2.16 | 19 | no | Muzzi A. et al., mSphere 2022; Viviani V. et al., NPJ Vaccines 2023 |
| M08389 | B | ST-162 complex | 162 | 2001 | USA | 22 | 14 | 2.21 | 20 | no | Muzzi A. et al., mSphere 2022; Viviani V. et al., NPJ Vaccines 2023 |
| M14569 | B | ST-35 complex | 35 | 2005 | USA | 22-1 | 14 | 2.16 | 21 | no | Muzzi A. et al., mSphere 2022; Viviani V. et al., NPJ Vaccines 2023 |
| M08129 | B | ST-41/44 complex/Lineage 3 | 44 | 2001 | USA | 7-4 | 1 | 2.19 | 144 | no | Muzzi A. et al., mSphere 2022 |
| M12898 | B | ST-35 complex | 457 | 2004 | USA | 5-1 | 2-2 | 2.16 | 143 | no | Muzzi A. et al., mSphere 2022; Viviani V. et al., NPJ Vaccines 2023 |
| M18711 | B | ST-35 complex | 35 | 2008 | USA | 22-1 | 14 | 2.16 | 339 | no | Muzzi A. et al., mSphere 2022 |
| LNP24651 | B | ST-32 complex | 32 | 2008 | FRA | 7 | 16-26 | 2.21 | 47 | yes | Viviani V. et al., NPJ Vaccines 2023 |
| ARG3753 | B | ST-865 complex | 3327 | 2014 | ARG | 21 | 16-36 | 2,119 | 24 | no | Efron A. et al., Hum Vaccin Immunother 2024 |
| ARG3191 | B | singlet | 12825 | 2011 | ARG | 21 | 16-36 | 2,119 | 24 | no | Efron A. et al., Hum Vaccin Immunother 2024 |
| ARG3175 | B | ST-865 complex | 3327 | 2011 | ARG | 21 | 16-36 | 2.new | 24 | no | Efron A. et al., Hum Vaccin Immunother 2024 |
| ARG3054 | B | ST-865 complex | 3327 | 2010 | ARG | 21 | 16-36 | 2,346 | 24 | no | Efron A. et al., Hum Vaccin Immunother 2024 |
| ARG3222 | B | ST-865 complex | 3327 | 2011 | ARG | 21 | 16-36 | 2,119 | 24 | no | Efron A. et al., Hum Vaccin Immunother 2024 |

**Supplementary Table 3: List of Neisseria meningitidis strains used in this work.** Information about the Clonal Complex, Sequence Type (ST), Year and Country of collection, PorA variant, variant or presence of 4CMenB antigens and Reference are reported for each strain.

| Strain alias | Year of collection | Country of collection | PorB variant | Reference |
|---|---|---|---|---|
| FA1090 | 1983 | / | PorB-1b | *Neisseria gonorrhoeae* (Zopf) Trevisan (ATCC 700825) |
| 1291 | 1972 | / | PorB-1b | *Neisseria gonorrhoeae* (Zopf) Trevisan (ATCC BAA-1839) |
| F62 | 1960 | / | PorB-1b | *Neisseria gonorrhoeae* (Zopf) Trevisan (ATCC BAA-1837) |
| SK92-679 | 1992 | / | PorB-1a | *Neisseria gonorrhoeae* (Zopf) Trevisan (ATCC BAA-1846) |
| WHO-N | 2001 | Australia | PorB-1a | HPE-England (NCTC 13482) |
| BG1 | 2014 | UK | PorB-1b | This study |
| BG5 | 2015 | UK | PorB-1b | This study |
| BG8 | 2015 | UK | PorB-1b | This study |
| BG9 | 2015 | UK | PorB-1b | This study |
| BG10 | 2013 | UK | PorB-1b | This study |
| BG11 | 2011 | UK | PorB-1a | This study |
| BG12 | 2011 | UK | PorB-1b | This study |
| BG13 | 2015 | UK | PorB-1b | This study |
| BG15 | 2015 | UK | PorB-1b | This study |
| BG19 | 2013 | UK | PorB-1b | This study |
| BG21 | 2015 | UK | PorB-1b | This study |
| BG22 | 2015 | UK | PorB-1b | This study |
| BG23 | 2013 | UK | PorB-1b | This study |
| BG24 | 2013 | UK | PorB-1b | This study |
| BG25 | 2015 | UK | PorB-1b | This study |
| BG26 | 2015 | UK | PorB-1b | This study |
| BG27 | 2013 | UK | PorB-1b | This study |
| BG29 | 2013 | UK | PorB-1b | This study |

**Supplementary Table 4: List of Neisseria gonorrhoeae strains used in this work.** Information about Year and Country of collection, PorB variant, variant and Reference are reported for each strain.

| mAb ID | Cluster | VH | JH | HCDR1 | HCDR2 | HCDR3 | VL | JL | LCDR1 | LCDR2 | LCDR3 |
|---|---|---|---|---|---|---|---|---|---|---|---|
| Sbj2_mAb13 | 1 | VH3-33 | IGHJ6 | GFTFSGSG | ISHDGSNK | AKDWAFTRRRGKEGFGFRTFYYYGMDV | VK1-9 | IGKJ4 | QGISSY | AAS | QHLNNYPLT |
| Sbj2_mAb6 | 2 | VH4-34 | IGHJ3 | GAPFSGPY | INFVGRT | ARGSIRYSSFPQGRRGDFDV | VK3-20 | IGKJ2 | QSVSSNY | SAS | QQYGSSPRFT |
| Sbj1_mAb11 | 2 | VH4-34 | IGHJ6 | GGSFSGYY | INNGGRS | ARMRVKAERFTFITPAVPTHYGMDV | VL1-51 | IGLJ2 | DSNIGSNS | END | ATWDNSLSAII |
| Sbj1_mAb18 | 2 | VH4-34 | IGHJ6 | GGSFSGYY | INNSGRS | ARMRVKAERFTFITPAVPTHYGMDV | VL1-51 | IGLJ1 | DSNIGSNS | END | ATWDNSLSAII |
| Sbj1_mAb20 | 2 | VH4-34 | IGHJ6 | GGSFSGYY | INYSGRS | ARMRVKAERFTFITPAVPTHYGMDV | VL1-51 | IGLJ7 | NSNIGSNS | DNN | GTWDNSLTAIV |
| Sbj1_mAb19 | 2 | VH4-34 | IGHJ6 | GGSFSGYY | INSSGRS | ARMRVKAERFTFITPSVPTHYGLDV | VL1-51 | IGLJ2 | DSNIGSNS | END | ATWDNSLSAII |
| Sbj1_mAb17 | 2 | VH4-34 | IGHJ6 | GGSFSGYY | INYSGRA | ARMRIKTERFTFITPAVPTHYGLDV | VL1-51 | IGLJ2 | DSNIGSNS | ENN | GTWDNSLRSII |
| Sbj1_mAb3 | 2 | VH4-34 | IGHJ6 | GGSFSGYH | IDYTGRS | ARMRIKTARFTFITPPFPGHYGLDV | VL1-51 | IGLJ2 | NSNIGSNS | DNT | ETWDNTLSAVV |
| Sbj3_mAb11 | 3 | VH1-18 | IGHJ4 | GYTFTSYG | ISAYNGNA | ARGRISSGWYGN | VK3-20 | IGKJ1 | QSVSSRY | GAS | QQYGNSPWP |
| Sbj3_mAb5 | 3 | VH1-69 | IGHJ1 | GGTFSNYA | IIPIFGTT | ARKGYRAALFHH | VK3-20 | IGKJ2 | QSVSSSY | GAS | QQYGSSPYT |
| Sbj2_mAb14 | 3 | VH1-69 | IGHJ6 | GGTFSNYA | ILPLYGTA | ARPQTLARYGMDV | VK3-20 | IGKJ4 | QSVSSNY | GAS | QQYGSSPLT |
| Sbj2_mAb19 | 3 | VH1-69 | IGHJ6 | GGTFSNYA | ILPLYGTA | ARPQTLARYGMDV | VL1-51 | IGLJ3 | GSNIGNNH | DNN | GTWDSSLSGGV |
| Sbj2_mAb4 | 3 | VH3-30 | IGHJ6 | GFTFRSYG | ISDDGSKK | AKDLSARRGYYYFYMDV | VL1-51 | IGLJ3 | SSNIGNNH | DNN | GTWDSSLSAGV |
| Sbj2_mAb15 | 3 | VH3-30 | IGHJ6 | GFTFRSYG | ISDDGSKK | AKDLSARRGYYYFYMDV | VL1-51 | IGLJ6 | SANIGNNH | DNN | GTWDSSLSAGV |
| Sbj2_mAb16 | 3 | VH3-33 | IGHJ6 | GFTFSAYG | ISDDGRSK | AKDLSARRGFYYFYMHV | VL1-51 | IGLJ3 | GSNIGNNH | DNN | GTWDSSLSGGV |
| Sbj2_mAb18 | 3 | VH3-23 | IGHJ6 | GFTFSSNA | IRGSGTTT | AKTLLSTRFKFVTSESKLFRAMDV | VL1-51 | IGLJ3 | SSNIGKSD | DNN | GTWDSSLSAGWV |
| Sbj3_mAb14 | 3 | VH4-30-4 | IGHJ6 | GGSISSGDYY | IYYSGNT | ARESRSDQSGGRKKYYYYFMDV | VK1-39 | IGKJ1 | QSVSSY | AAS | QQSYSSKT |
| Sbj3_mAb8 | 3 | VH3-21 | IGHJ4 | GFTFSSHS | ISGGSSSSYT | VRWRSARVTGRSSLSRGAGVGSFDY | VK1-39 | IGKJ2 | QSISSY | AAS | QQSYSTPRT |

**Supplementary Table 5: HumAbs sequence data annotation.** The Gene Family of the Variable (V) and Joining (J) region and the aminoacidic sequence of the 3 Complementarity-determining regions (CDRs) of the Heavy (H) and Light (L) chains are reported for each HumAb.
